# Whole blood count data and hematological parameters in US vegetarians: a cross-sectional study

**DOI:** 10.1007/s00394-025-03778-8

**Published:** 2025-09-18

**Authors:** Maximilian Andreas Storz, Stefanie Kowarschik, Julian Herter, Roman Huber

**Affiliations:** Department of Internal Medicine II, Centre for Complementary Medicine, Medical Center – University of Freiburg, Faculty of Medicine, University of Freiburg, Freiburg, Germany

**Keywords:** Lacto-ovo-vegetarian, White blood cell count, Lymphocytes, Platelets, Nutrition, Epidemiology

## Abstract

**Purpose:**

Lower white blood cell, neutrophil and lymphocyte counts have been reported in vegetarians. However, studies on this topic revealed conflicting results and most observations stem from small studies using convenience sampling techniques. Additional large studies investigating associations between vegetarian diets and whole blood count (WBC) data are thus warranted.

**Methods:**

We examined WBC and hematological parameters in US vegetarians based on complex, multistage, probability sampling design-based data from the cross-sectional National Health and Nutrition Examination Surveys (NHANES). Crude and multivariable regression-adjusted cell counts were compared between lacto-ovo-vegetarians (LOVs), semi-vegetarians (SVGs) and omnivores.

**Results:**

The analysis included *n* = 8232 unweighted observations, thereof *n* = 98 LOVs and *n* = 110 SVGs. Vegetarians were characterized by a younger age, a healthier BMI, and a higher proportion of females. Crude and adjusted white blood cell counts were lower in LOVs but did not differ significantly from omnivores. Except for lower red blood cell counts and hemoglobin levels in vegetarians, no significant between group differences in WBC data were found. White blood cell counts correlated significantly with the BMI and selenium intake in LOVs (*r* = 0.27 for both). Each additional µg of dietary vitamin B12 was associated with an increase in white blood cells by 0.11*1000 cells/µl in vegetarians.

**Conclusion:**

While hemogram alterations were directionally consistent with previous studies, differences in white blood cells and neutrophils were not significant between NHANES vegetarians and omnivores. Statistical and design-specific aspects as well the elevated BMI in the vegetarian subpopulation could serve as potential explanations.

**Supplementary Information:**

The online version contains supplementary material available at 10.1007/s00394-025-03778-8.

## Introduction

Alterations in white blood cell levels and other whole blood count parameters in vegetarians and vegans as well as their potential underlying mechanisms have long been subject to a scientific debate [1]. Several studies suggested that vegetarian diets (which exclude animal flesh but include dairy and eggs [2]) and vegan diets (which exclude all animal foods [2]) may have an impact on platelets, as well as white and red blood cell counts [3, 4].

A 2019 study based on the large UK biobank dataset revealed significantly lower counts in age- and sex-adjusted total white blood cells, neutrophils, lymphocytes, and monocytes in white British vegetarians as compared to regular meat eaters [5]. Compared with regular meat eaters, non- or low-meat eaters also had lower hemoglobin concentrations. Mean platelet counts were lowest in vegans and highest in vegetarians [5]. An American study investigating the nutritional status of Loma Linda university students also revealed significantly lower lymphocyte, platelet and white blood cell counts in vegans, although no differences were found with regard to the neutrophil count [3]. Our own nutrition intervention studies pointed at a reduction in neutrophils, monocytes and platelets upon a one month vegan dietary intervention [4].

Higher white blood cell counts have been proposed as an early marker of inflammation [5–7]. The observed lower white blood cell counts in vegetarians and vegans could thus be interpreted as an anti-inflammatory effect of these diets [4, 5, 8]. The lower counts of other potentially pro-inflammatory cells in vegans (e.g., platelets) further support this hypothesis. On the other hand, low white blood cell counts could also be an indicator for an impaired immune system or a bone marrow impairment [5], e.g., due to diet-induced nutritional deficiencies, including vitamin B12 [9], zinc [3], or iron deficiency [10].

The associations between plant-based dietary patterns and lower white blood cell counts (and the underlying mechanisms) are still poorly understood [4]. Overall, there is a paucity of studies that reported whole blood count data in different vegetarian and vegan populations [5]. With the exception of the UK biobank study, most studies were small and included ≤ 100 participants [5]. A better understanding in larger cohorts, however, is urgently warranted, as the underlying mechanisms could be relevant for the lower chronic disease risk in individuals consuming a plant-based diet [5].

To address this gap in the literature, we investigated whole blood count data in vegetarians in the US-based National Health and Nutrition Examination Surveys (NHANES). The objectives were twofold: to investigate potential associations between vegetarian status and whole blood count data, and to identify nutrients associated with lower cell counts. This study was performed under the hypothesis that US vegetarians would be characterized by lower levels of white blood cells, platelets and lymphocytes in comparison to the US general population.

## Materials and methods

### Study population and design

The NHANES is a nationally representative, cross-sectional survey of the non-institutionalized US population with publicly available data [11–14]. NHANES is based on complex, stratified, multistage probability cluster sampling design data and allows for large-scale epidemiological analyses [15, 16]. Each NHANES cycle surveys about *n* = 5000 participants [13]. All participants provide written informed consent. Additional information regarding the NHANES may be obtained from the official website [11].

### Vegetarian status assessment

The vegetarian status was drawn from the NHANES diet behavior and nutrition module [17]. This module included the question “Do you consider yourself to be a vegetarian?” which was used for the initial group assignment. Participants who replied with “Yes” were considered vegetarians, whereas those replying “No” were considered to belong to the general population. Participants who replied with “Don’t know” were not considered for this analysis. Vegetarian status assessment was thus initially based on a self-reported binary question.

Notably, the diet behavior of NHANES vegetarians has been analyzed in the past, and a previous study by Juan et al. suggested that several self-identified NHANES vegetarian reported the consumption of some type of flesh products (meat, poultry, and/or seafood) [18]. Taking these findings into account, we constructed two further subgroups and divided the self-identified vegetarian group in a “lacto-ovo-vegetarian” group and “semi-vegetarian” group. For this, we considered individual food intake data from a 24-h dietary recall. Participants who self-identified as vegetarians and who consumed no meat, poultry, and seafood were considered “lacto-ovo-vegetarians”. Those who self-identified as vegetarians but indicated the consumption of animal flesh in the dietary recall were considered “semi-vegetarians”. Individuals who did not consider themselves vegetarians and who reported the consumption of animal flesh in the 24-h dietary recall were assigned to the general population. We refrained from constructing a fourth group (pesco-vegetarians) in light of the already small sample size and with regard for the need to construct groups for which reliable subpopulation statistics could be computed (see statistical analysis below for details). A vegan group was not built due to the very low number of observations (*n* < 5).

### Primary outcome

The primary outcome for this epidemiological analysis was the (crude and adjusted) white blood cell count (in 1000 cells/µL) in the vegetarian subgroups in comparison to the general population. Whole blood samples were analyzed in the NHANES mobile examination centers (MEC) [19]. Detailed specimen collection and processing instructions are available from the NHANES Laboratory/Medical Technologists Procedures Manual [20]. In brief, the methods used to derive the complete blood count parameters were based on the Beckman Coulter method of counting and sizing, in combination with an automatic diluting and mixing device for sample processing, and a single beam photometer for hemoglobinometry [19]. The white blood cell differential used the “Volume, Conductivity, and Scatter“ technology. The NHANES emphasized that there were no changes to the lab methods or lab site between the 2007/2008 and the 2009/2010 cycle [19]. White blood cells were selected as the primary outcome for their known association with systemic inflammation [21], and in light of previous studies reporting lower white blood cell levels in US and Polish vegetarians [3, 22].

### Secondary outcomes

Secondary outcomes of interest included the platelet count, the neutrophil count and the lymphocyte count. Platelets play an important role in immune-mediated inflammatory diseases [23], and higher platelet levels and lower volumes in vegetarians have been reported previously in British white vegetarians [5]. The aforementioned secondary outcomes were also selected in light of a recent Chinese study that suggested an association between vegetarian status and the neutrophil to lymphocyte ratio [24].

### Other whole blood count data of interest

Further to that, we also investigated other complete blood count data including hemoglobin levels and the hematocrit, which were also provided in the NHANES full blood count data set. Notably, marginal predicted values were not estimated for these remaining parameters.

### Sociodemographic data and covariates

A large set of sociodemographic variables was included to characterize the study population in great detail, including sex (binary categorical variable: male, female), age (continuous variable), race/ethnicity (categorical variable: Mexican Americans, Other Hispanic, Non-Hispanic White, Non-Hispanic-Black, Other Race), educational level (categorical variable: less than 9th grade, 9–11th grade, high school graduate, some college or associate degree, college graduate or above), and marital status (categorical variable: living with a partner/married, divorced/separated/widowed, never married).

As for the lifestyle factors, we included alcohol intake (binary categorical variable: ≥12 or < 12 drinks per year), smoking status (categorical variable: non-smoker, current smoker, former smoker), physical activity (continuous variable: minutes of sedentary time per day), history of recent head or chest cold (binary categorical variable: yes, no), history of recent flu, pneumonia or ear infection (binary categorical variable: yes, no) and body mass index (presented as a continuous variable and as a categorical variable with 4 categories). Nutrient intakes were obtained based on 24-h dietary recall data [25].

Apart from sociodemographic and lifestyle-related covariates, we also added laboratory markers. Beyond the complete blood count data [19], we also added folate serum concentrations as well as red blood cell folate (nmol/L) and C-reactive protein levels (mg/dl) as all three variables were deemed important with regard to the primary hypothesis of the study.

### Inclusion and exclusion criteria

Only NHANES participants with a complete dataset were considered. Participants with missing data on any variable of interest were excluded. In addition to that, we pre-specified the following exclusion criteria: implausible energy intake/dietary data (as defined by an energy intake < 600 or > 5000 kcal/d), severe leukopenia or leukocytosis (as defined by a white blood cell count < 3000 or > 14000 cells/µl), severe lymphopenia or lymphocytosis (as defined by a lymphocyte count < 500 or > 6000 cells/µl), and severe thrombocytopenia or thrombocytosis (as defined by a platelet count < 100000 or > 500000 cells/µl). The rationale behind this approach was twofold: (I) to exclude NHANES participants with known or unknown hematological disorders, and (II) to construct a cohort with healthy individuals with blood count data within the normal range in order to minimize the impact of potential confounders.

### Statistical analysis

The statistical analysis was performed in Stata Statistical Software (StataCorp (2015). Statistical Software: Release 14. College Station, TX: StataCorp LP). Visualization was performed in Stata 19 (StataCorp. 2025. Stata Statistical Software: Release 19. College Station, TX: StataCorp LLC). Two consecutive NHANES cycles were merged (2007–2008 and 2009–2010), which both included the ‘vegetarian status’ variable (‘DBQ915’) [17, 26, 27]. NHANES cycles prior to 2007 and after 2010 did not include this particular variable, and were thus not considered. A 4-year weight for medical examination center data was subsequently constructed (wtmec4year = wtmec2year/2), taking into account the NHANES tutorial weighting module and the analytic guidelines [28]. To account for the complex NHANES survey design characteristics and population weights, we performed weighted survey analyses throughout the entire analysis process using Stata’s “svyset” and “svy” commands.

In a second step, we defined the subpopulations to examine by excluding participants with missing data for any variable of interest. No imputation procedures were performed. The statistical analysis was performed in accordance with the recommendations of West, Berglund, and Heeringa for applied survey data analyses [29].

Descriptive statistics were estimated using subpopulation commands. Sociodemographic, anthropometric, laboratory and nutrient intake data were compared between the general population and vegetarians. Subpopulation statistics were additionally computed for lacto-ovo-vegetarians and semi-vegetarians. Data distribution was examined via histograms, box plots and subpopulation summary statistics. To create histograms, we followed the University of California’s “Survey Data Analysis in Stata” tutorial and used the integer part of the sampling weight by creating a frequency weight from the sampling weight [30]. Normally distributed data was described with the mean and corresponding 95%-confidence interval (CI).

For categorical variables, we provided weighted proportions with their 95%-confidence intervals. Prior analyses in NHANES vegetarians already revealed a low number of unweighted observations in the vegetarian subgroup [31–33]. All weighted proportions were thus checked for reliability with utmost care [34]. Our approach has been described previously and covered a careful comparison to the 2017 NCHS (National Center for Health Statistics) data presentation standards for proportions [34, 35]. Korn-Graubard CIs, the CI width, the sample size, and the degrees of freedom were considered upon assessing the reliability of a weighted proportion. To assess whether the NCHS criteria were met, we used Ward’s user-written command “kg_nchs” [36].

In a subsequent step, we estimated correlations between blood cell types and various anthropometric/dietary variables in the vegetarian subpopulation as well as their level of significance based on an approach described earlier by Sribney [37].

We then constructed multivariable linear regression models based on an approach by Heeringa et al. [29]. First, we conducted bivariate exploratory analyses beyond the aforementioned correlations and a literature search to identify potential candidate predictors for the outcomes of interest (white blood cells, platelets, lymphocytes and neutrophils). Clinically and/or scientifically relevant predictors with a bivariate relationship of significance *p* < 0.25 with the outcome variable were included in the initial model. Vegetarian status was included regardless of the level of significance. A basic set of four covariates was included in all models, including age, sex and ethnicity/race and vegetarian status. In subsequent models, additional covariates were individually added to this basic set (energy intake; body mass index; alcohol intake and smoking status; selenium intake; vitamin B12 intake; vitamin A intake; zinc intake). The final model included all previously added covariates together in a singular model. Nine models were constructed for each outcome of interest. The aforementioned covariates purposefully included potential mediators, e.g., smoking, which is less prevalent in vegetarians. Model improvement strategies such as interactions and adding square terms (to investigate potential quadratic relationships) were then tested.

In a subsequent step, we used Stata’s ‘margins’ function to display marginal predicted values of the outcome of interest for each level of the vegetarian status (e.g., non-vegetarian vs semi-vegetarian vs lacto-ovo-vegetarian) at all possible increments of 10 years (from 20 to 80). We then plotted these marginal predicted values including their 95% confidence intervals using Stata’s ‘marginsplot’. The post-estimation command ‘contrasts’ was then used to test linear hypotheses and to form contrasts involving factor variables from all fitted models. Both differences from a reference level (‘r.-operator’ contrasts with non-vegetarians as the reference group) and from the grand mean (‘gw.-operator’, a weighted contrast operator) were tested. Alpha was set to 0.05.

All dietary variables (protein, fat, vitamin A, vitamin B12, zinc, selenium phosphorus, iron and fiber) were then added in a final multivariable regression model to estimate white blood cell counts (and, in separate models, platelet, lymphocyte and neutrophil counts) for the entire sample and for the self-identified vegetarian subsample. All models additionally adjusted for age, sex, race/ethnicity, and smoking status. Jann’s ‘coefplot‘ function was used to plot the regression coefficients of interest [38].

## Results

Total number of unweighted observations in the present study was *n* = 8,232. The sample included a total of *n* = 208 vegetarians, thereof *n* = 98 lacto-ovo-vegetarians and *n* = 110 semi-vegetarians. Supplementary Fig. 1 depicts a participant inclusion flowchart.

Table 1 shows the sample’s characteristics including sociodemographic data by vegetarian status. Vegetarian status was not independent from sex, ethnicity and educational level (*p* < 0.001). The weighted proportion of females among vegetarians was significantly higher as compared to the general population. The data also suggested a significantly larger weighted proportion of college graduates among vegetarians. Vegetarian subgroup analyses (semi-vegetarians vs lacto-ovo-vegetarians) were subsequently performed and are also shown in Table 1. Notably, due to the overall low number of unweighted observations in the vegetarian group (*n* = 208), many proportions had to be flagged as unreliable.

**Table 1:** Sample characteristics by vegetarian status

	General population (n = 8024)	Vegetarians (n = 208)	*p*-value	Semi-vegetarian (n = 110)	Lacto-ovo-vegetarian (n = 98)
Sex			*p* < 0.001^b^		
Male	49.97% (49.04–50.91)	32.74% (25.94–40.34)**		28.41% (16.98–43.49)	35.69% (24.85–48.23)
Female	50.03% (49.09–50.96)	67.26% (59.66–74.06)**		71.59% (56.51–83.02)	64.31% (51.77–75.15)
Age (years)	47.10 (46.37–47.84)	44.65 (41.11–48.19)	*p* = 0.161^c^	49.72 (46.18–53.26)	41.19 (36.96–45.42)
Ethnicity/race			*p* < 0.001^b^		
Mexican American	8.53% (6.10–11.82)	6.94% (4.23–11.19)		9.75% (5.02–18.07)*	5.02% (2.56–9.63)*
Other Hispanic	4.94% (3.39–7.16)	5.04% (3.05–8.21)*		9.12% (5.46–14.84)*	2.25% (0.74–6.62)*^,^**
Non-Hispanic White	69.92% (64.51–74.83)	61.55% (46.20–74.90)*		52.67% (40.66–64.37)	67.63% (44.60–84.42)*
Non-Hispanic Black	10.94% (8.89–13.40)	7.09% (4.35–11.34)**		13.00% (7.31–22.08)	3.05% (1.17–7.70)*^,^**
Other Race^a^	5.66% (4.51–7.10)	19.38% (9.23–36.23)*;**		15.46% (7.60–28.90)*	22.05% (8.00–47.92)*
Education level			*p* = 0.001^b^		
Less than 9th grade	6.14% (5.16–7.30)	9.12% (5.42–14.93)		15.78% (9.41–25.27)	4.56% (2.18–9.28)*,**
9–11th grade	12.43% (11.04–13.98)	8.78% (5.67–13.36)		12.08% (7.24–19.48)	6.53% (2.73–14.79)*
High school graduate/GED	24.52% (22.84–26.29)	12.18% (6.14–22.72)*,**		12.01% (5.38–24.67)*	12.30% (5.48–25.34)*
Some college or AA degree	30.05% (28.48–31.66)	27.38% (20.85–35.05)		39.49% (27.14–53.35)	19.11% (11.62–29.78)**
College graduate or above	26.86% (24.08–29.83)	42.54% (32.47–53.28)**		20.64% (13.63–30.00)	57.51% (42.59–71.17)*,**
Marital status			*p* = 0.227^b^		
Living with a partner/married	65.00% (62.76–67.17)	56.32% (45.14–66.89)		59.75% (47.58–70.83)	53.97% (40.97–66.47)
Divorced/separated/widowed/	17.99% (16.86–19.18)	22.26% (15.09–31.57)		25.87% (16.16–38.71)	19.80% (11.53–31.85)
Never married	17.01% (15.37–18.78)	21.42% (13.41–32.43)		14.38% (8.68–22.89)	26.23% (14.86–41.99)
≥ 12 alcoholic drinks per year			*p* = 0.001^b^		
No	22.69% (20.79–24.71)	34.26% (27.26–42.01)**		37.23% (26.48–49.42)	32.23 (22.93–43.19)
Yes	77.31% (75.29–79.21)	65.74% (57.99–72.74)**		62.77% (50.58–73.52)	67.77 (56.81–77.07)
Smoking status			*p* = 0.064^b^		
Non-smoker	53.62% (51.36–55.87)	64.45% (51.66–75.47)		68.29% (55.84–78.58)	61.83% (43.74–77.15)*
Current smoker	25.28% (23.79–26.82)	24.44% (17.36–33.25)		23.20% (15.20–33.73)	25.29% (14.88–39.60)
Former smoker	21.10% (19.45–22.86)	11.10% (6.03–19.56)**		8.51% (4.18–16.53)*	12.88% (5.98–25.55)*
Minutes sedentary activity/day	339.25 (330.15–348.35)	319.03 (280.37–357.69)	*p* = 0.314^c^	225.95 (201.47–250.44)	383.03 (331.99–434.08)
History of recent head or chest cold			*p* = 0.574^b^		
Yes	15.56% (13.85–17.44)	17.78% (10.65–28.19)		15.30% (7.74–28.00)*	19.48% (10.66–32.91)
No	84.44% (82.56–86.15)	82.22% (71.81–89.35)		84.70% (72.00–92.26)*	80.52% (67.09–89.35)
History of recent flu, pneumonia or ear infection			*p* = 0.649^b^		
Yes	3.73% (3.16–4.39)	3.10% (1.35–6.93)*		3.55% (1.24–9.74)*	2.79% (0.80–9.26)*
No	96.27% (95.61–96.84)	96.90% (93.07–98.65)*		96.45% (90.26–98.76)*	97.21% (90.74–99.20)*
BMI (kg/m^2^)	28.89 (28.71–29.08)	25.80 (24.87–26.74)	*p* < 0.001^c^	27.00 (25.89–28.11)	24.99 (23.81–26.16)
BMI category			*p* < 0.001^b^		
< 18.50	1.47% (1.11–1.93)	4.52% (1.61–12.02)*		3.05% (0.49–16.75)*	5.52% (1.65–16.88)*
≥ 18.50 & <25.00	28.01% (26.47–29.59)	45.68% (37.69–53.90)**		41.55% (30.59–53.42)	48.51% (38.03–59.12)
≥ 25.00 & <30.00	34.47% (32.89–36.10)	31.95% (24.51–40.44)		27.37% (17.58–39.95)	35.08% (24.93–46.80)
≥ 30	36.05% (34.58–37.55)	17.85% (11.92–25.85)**		28.03% (19.15–39.04)	10.89% (5.25–21–25)*,**

Weighted proportions. Total number of unweighted observations: *n* = 8,232. The sample included *n* = 208 vegetarians, thereof *n* = 98 lacto-ovo-vegetarians and *n* = 110 semi-vegetarians. Continuous variables shown as mean (95%-CI). Categorical variables shown as weighted proportion (95%-CI)

GED = General Equivalency Diploma

* indicates an unreliable proportion, as per recent NCHS Guidelines

** indicates significant differences in weighted proportions

^a^ = includes Multi-Racial

^b^ = based on Stata’s design-adjusted Rao–Scott test

^c^ = based on regression analyses followed by adjusted Wald tests

The between group difference in BMI was significantly different when comparing the general population with the lacto-ovo-vegetarian subgroup and the semi-vegetarian subgroup, respectively, (*p* < 0.001 for both)

The mean BMI of vegetarians was significantly lower in self-identified vegetarians when compared to the general population. Still, the mean BMI in the vegetarian group was 25.80 (24.87–26.74) kg/m^2^ and thus not within a normal range. The proportion of participants who indicated consumption of ≥ 12 alcoholic drinks per year was also significantly lower in vegetarians, however, no significant differences were found with regard to smoking. The share of participants with a recent flu, pneumonia or ear infection was negligible in both groups.

Table 2 summarizes the results of the whole blood count analysis by vegetarian status. Crude analyses showed no significant between group differences in whole blood count data, except for the red blood cell count as well as hemoglobin and hematocrit levels. All 3 parameters were significantly lower in vegetarians, although difference appeared marginal for red blood cells and the hematocrit. For additional insights, Supplementary Fig. 2 additionally depicts histograms showing the distribution of the while blood cell count, lymphocyte count, neutrophil count and platelet count in the whole sample.

**Table 2:** Complete blood count data and other laboratory values by vegetarian status

	General population (n = 8024)	Vegetarians (n = 208)	*p*-value	Semi-vegetarian (n = 110)	Lacto-ovo-vegetarian (n = 98)	*p*-value
*Complete blood count data*						
White blood cell count (1000 cells/µl)	7.16 (7.07–7.25)	6.97 (6.56–7.37)	*p* = 0.365^a^	7.06 (6.54–7.58)	6.90 (6.31–7.49)	*p* = 0.663^b^
Lymphocyte count (1000 cells/µl)	2.11 (2.08–2.13)	2.13 (2.02–2.25)	*p* = 0.633^a^	2.19 (2.04–2.35)	2.09 (1.93–2.25)	*p* = 0.521^b^
Monocyte count (1000 cells/µl)	0.55 (0.54–0.56)	0.52 (0.49–0.55)	*p* = 0.054^a^	0.51 (0.47–0.55)	0.53 (0.49–0.56)	*p* = 0.128^b^
Eosinophil count (1000 cells/µl)	0.20 (0.20–0.21)	0.19 (0.17–0.22)	*p* = 0.540^a^	0.17 (0.14–0.19)*	0.21 (0.18–0.25)	*p* = 0.029^b^
Basophil count (1000 cells/µl)	0.04 (0.04–0.04)	0.04 (0.03–0.05)	*p* = 0.803^a^	0.04 (0.03–0.05)	0.04 (0.01–0.05)	*p* = 0.947^b^
Neutrophil count (1000 cell/µl)	4.26 (4.20–4.33)	4.07 (3.74–4.40)	*p* = 0.269^a^	4.15 (3.73–4.56)	4.02 (3.58–4.47)	*p* = 0.541^b^
Red blood cell count (million cells/µl)	4.68 (4.65–4.71)	4.55 (5.48–4.63)	*p* = 0.001^a^	4.48 (4.34–4.61)*	4.61 (4.50–4.71)	*p* = 0.002^b^
Hemoglobin (g/dL)	14.34 (14.23–14.46)	13.85 (13.60–14.10)	*p* < 0.001^a^	13.71 (13.28–14.15)*	13.95 (13.68–14.22)*	*p* < 0.001^b^
Hematocrit (%)	41.65 (41.36–41.95)	40.32 (39.61–41.02)	*p* < 0.001^a^	40.01 (38.76–41.25)*	40.53 (39.71–41.35)*	*p* < 0.001^b^
Mean cell volume (fL)	89.24 (88.81–89.67)	88.76 (87.89–89.64)	*p* = 0.291^a^	89.60 (88.22–90.99)	88.19 (87.02–89.37)	*p* = 0.226^b^
Mean cell hemoglobin (pg)	30.73 (30.53–30.93)	30.50 (30.10–30.90)	*p* = 0.271^a^	30.72 (30.13–31.30)	30.35–29.82–30.89)	*p* = 0.441^b^
MCHC (g/dL)	34.42 (34.28–34.56)	34.34 (34.11–34.56)	*p* = 0.449^a^	34.25 (34.02–34.48)	34.40 (34.08–34.71)	*p* = 0.236^b^
Platelet count (1000 cells/µl)	252.61 (249.81–255.41)	260.17 (251.69–268.66)	*p* = 0.122^a^	262.19 (248.69–275.69)	258.80 (246.26–271.33)	*p* = 0.253^b^
Red cell distribution width (%)	12.74 (12.69–12.79)	12.66 (12.48–12.84)	*p* = 0.423^a^	12.74 (12.52–12.97)	12.60 (12.33–12.87)	*p* = 0.660^b^
*Other laboratory values*						
C-reactive protein (mg/dL)	0.38 (0.36–0.40)	0.36 (0.17–0.54)	*p* = 0.780^a^	0.37 (0.25–0.49)	0.35 (0.06–0.63)	*p* = 0.958^b^
Red blood cell folate (nmol/L)	1206.79 (1172.22–1241.36)	1342.72 (1250.52–1434.91)	*p* = 0.007^a^	1320.77 (1153.15–1488.39)	1357.71 (1235.18–1480.23)*	*p* = 0.027^b^
Serum folate (nmol/L)	43.25 (41.92–44.59)	53.43 (47.73–59.13)	*p* = 0.002^a^	48.73 (40.30–57.17)	56.64 (48.69–64.58)*	*p* = 0.005^b^

Based on *n* = 8,232 observations. The sample included *n* = 208 vegetarians, thereof *n* = 98 lacto-ovo-vegetarians and *n* = 110 semi-vegetarians. Continuous variables shown as mean (95%-CI)

Significant differences in pairwise comparisons (e.g., lacto-ovo-vegetarians vs general population; semi-vegetarians vs general population) were marked with a “*” symbol

^a^ = *p* for the difference between the general population and the vegetarian subpopulation

^b^ = *p* for overall heterogeneity

Significant between group differences were found for the intakes of many nutrients (Table 3). Energy intake was significantly lower in self-identified vegetarians (1913.88 (1776.14–2051.62) vs 2177.03 (2146.33–2207.33) kcal/d). Nevertheless, self-identified vegetarians consumed higher total amounts of fiber, carbohydrates, magnesium and folic acid. Furthermore, we estimated a lower intake of total fat, saturated fat, cholesterol, selenium, alcohol and protein in vegetarians in comparison to the general population.

**Table 3:** Nutrient intake data by vegetarian status

	General population (n = 8024)	Vegetarians (n = 208)	*p*-value	Semi-vegetarian (n = 110)	Lacto-ovo-vegetarian (n = 98)	*p*-value
Energy intake (kcal/d)	2177.03 (2146.33–2207.33)	1913.88 (1776.14–2051.62)	*p* = 0.000^a^	1909.30 (1657.64–2160.97)*	1917.00 (1733.49–2100.50)*	*p* = 0.002^b^
Fiber intake (g/d)	16.15 (15.62–16.69)	21.85 (19.58–24.13)	*p* < 0.001^a^	18.52 (15.23–21.80)	24.13 (20.59–27.67)*	*p* < 0.001^b^
*Macronutrients and fats*						
Carbohydrate intake (g/d)	258.02 (254.16–261.88)	268.38 (246.33–290.44)	*p* = 0.338^a^	247.15 (216.54–277.75)	282.89 (246.20–319.58)	*p* = 0.384^b^
Protein intake (g/d)	86.00 (84.54–87.45)	62.14 (56.93–67.35)	*p* < 0.001^a^	69.96 (60.15–79.77)*	56.80 (51.94–61.66)*	*p* < 0.001^b^
Fat intake (g/d)	82.94 (81.38–84.51)	64.65 (58.44–70.86)	*p* < 0.001^a^	70.13 (57.87–82.40)*	60.91 (53.50–68.31)*	*p* < 0.001^b^
Saturated fat intake (g/d)	27.08 (26.42–27.74)	20.29 (18.31–22.26)	*p* < 0.001^a^	20.98 (17.34–24.61)*	19.81 (16.94–22.69)*	*p* < 0.001^b^
Monounsaturated fat intake (g/d)	30.44 (29.88–31.00)	22.96 (20.30–25.61)	*p* < 0.001^a^	26.58 (21.42–31.74)	20.48 (17.57–23.39)*	*p* < 0.001^b^
Polyunsaturated fat intake (g/d)	18.01 (17.66–18.37)	16.17 (14.39–17.94)	*p* = 0.042^a^	16.91 (13.58–20.24)	15.66 (13.72–17.60)*	*p* = 0.061^b^
*Micronutrients, vitamins and others*						
Alcohol intake (g/d)	11.55 (10.42–12.69)	6.73 (3.73–9.73)	*p* = 0.003^a^	5.76 (2.43–9.08)*	7.40 (2.45–12.34)	*p* = 0.002^b^
Calcium intake (mg/d)	965.27 (941.64–988.90)	939.81 (856.05–1023.57)	*p* = 0.530^a^	839.74 (699.31–980.17)	1008.15 (898.45–1117.86)	*p* = 0.185^b^
Cholesterol (mg/d)	303.59 (296.13–311.05)	166.19 (128.48–203.90)	*p* < 0.001^a^	211.89 (152.58–271.21)*	134.98 (93.99–175.98)*	*p* < 0.001^b^
Choline intake (mg/d)	348.32 (342.32–354.33)	244.17 (220.02–268.32)	*p* < 0.001^a^	263.42 (216.28–310.57)*	231.02 (211.50–250.54)*	*p* < 0.001^b^
Folic acid intake (µg/d)	186.38 (179.74–193.02)	206.39 (178.85–233.93)	*p* = 0.109^a^	158.61 (127.66–189.55)	239.03 (199.83–278.22)*	*p* = 0.014^b^
Iron intake (mg/d)	15.45 (15.08–15.83)	15.42 (13.82–17.03)	*p* = 0.968 ^a^	13.94 (11.81–16.08)	16.43 (14.39–18.47)	*p* = 0.217 ^b^
Magnesium intake (mg/d)	301.96 (294.90–309.12)	319.67 (286.82–352.51)	*p* = 0.281^a^	297.22 (235.93–358.51)	334.99 (294.01–375.98)	*p* = 0.321^b^
Phosphorus intake (mg/d)	1403.34 (1380.50–1426.18	1194.33 (1099.70–1288.96)	*p* < 0.001 ^a^	1185.12 (1012.19–1358.06)*	1200.62 (1089.15–1312.10)*	*p* < 0.001^b^
Potassium intake (mg/d)	2760.09 (2712.77–2807.41)	2564.35 (2352.38–2776.31)	*p* = 0.068^a^	2523.02 (2152.52–2893.53)	2592.57 (2336.90–2848.24)	*p* = 0.194^b^
Selenium (µg/d)	115.30 (112.98–117.63)	92.14 (82.67–101.62)	*p* < 0.001^a^	95.45 (81.04–109.86)*	89.89 (80.08–99.69)*	*p* < 0.001^b^
Vitamin A, RAE (µg/d)	622.12 (601.93–642.32)	686.31 (560.07–812.56)	*p* = 0.294^a^	604.53 (460.28–748.78)	742.17 (594.59–889.74)	*p* = 0.204^b^
Vitamin B12 intake (µg /d)	5.65 (5.47–5.83)	3.64 (3.14–4.14)	*p* < 0.001^a^	3.94 (3.14–4.74)*	3.44 (2.95–3.93)*	*p* < 0.001^b^
Zinc (mg/d)	12.50 (12.12–12.88)	8.72 (8.02–9.42)	*p* < 0.001^a^	8.79 (7.55–10.02)*	8.67 (7.85–9.50)*	*p* < 0.001^b^

Total number of unweighted observations: *n* = 8,232. The sample included *n* = 208 vegetarians, thereof *n* = 98 lacto-ovo-vegetarians and *n* = 110 semi-vegetarians. Continuous variables shown as mean (95%-CI)

Significant differences in pairwise comparisons (e.g., lacto-ovo-vegetarians vs general population; semi-vegetarians vs general population) were marked with a “*” symbol

RAE = Retinol Activity equivalents

^a^ = *p* for the difference between the general population and the vegetarian subpopulation

^b^ = *p* for overall heterogeneity

In a subsequent step, we investigated potential bivariate associations between the four cell types of interest (white blood cells, platelets, lymphocytes and neutrophils) and various other anthropometric and dietary variables. For this, scatterplots were constructed (Supplementary Figs. 3–6) and Pearson’s product-moment correlations were run. Weak but significant positive correlations were found for the body mass index and the white blood cell count in lacto-ovo-vegetarians as well as for dietary selenium intake and the white blood cell count (*r* = 0.27, *p* = 0.045 and *r* = 0.27, *p* = 0.022, respectively). As for the platelet count, we observed a significant inverse correlation with the total energy intake (*r*= − 0.20, *p* = 0.001) and the fiber intake (*r*= − 0.23, *p* = 0.007). Neutrophiles correlated significantly with the body mass index (*r* = 0.29, *p* = 0.012) in lacto-ovo-vegetarians. In semi-vegetarians, we found a moderate positive correlation between the vitamin B12 intake (in µg/d) and the neutrophil count (*r* = 0.37, *p* = 0.030). Associations between dietary fat intakes and the four cell types of interest are separately depicted in Supplementary Figs. 7–10. Significant inverse correlations were found between the platelet count and the total fat intake (*r*= − 0.19, *p* = 0.038), the monounsaturated fat intake (*r*= − 0.25, *p* = 0.018), and the polyunsaturated fat intake (*r*= − 0.26, *p* = 0.004) in lacto-ovo-vegetarians.

**Fig. 1:**
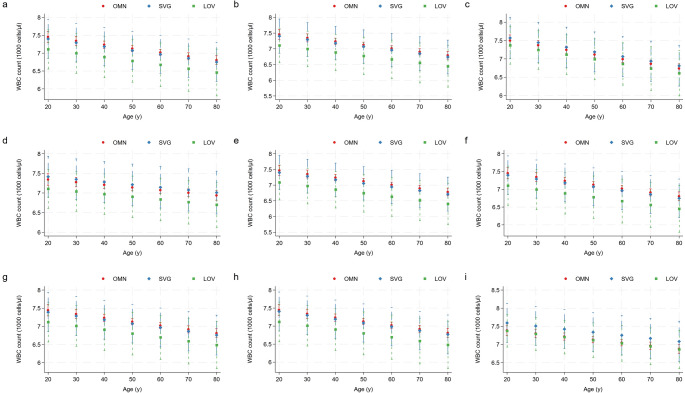
Margins plot—white blood cell count by vegetarian status. Plots of marginal predicted values (adjusted means) based on a series of regression models, illustrating differences in the relationship of the white blood cell count (in 1000 cells/µl) and age, depending on the vegetarian status. The general population (OMN – omnivores) is shown in red, whereas semi-vegetarians (SVG) are shown in blue and lacto-ovo-vegetarians (LOV) are shown in green. In panel **a**, we adjusted for age, sex and ethnicity. In addition to these 3 variables, panels**b**–**h** included the following adjustments: **b**: additional adjustment for energy intake (kcal/d); **c**: additional adjustment for the body mass index (kg/m^2^), **d**: additional adjustment for alcohol intake and smoking status; **e**: additional adjustment for selenium intake; **f**: additional adjustment for vitamin B12 intake; **g**: additional adjustment for vitamin A intake; **h** additional adjustment for zinc intake. In panel **i**, we adjusted for all aforementioned covariates in a singular model

Figure 1 displays plots of marginal predicted values (adjusted means) based on a series of regression models, illustrating differences in the relationship of the white blood cell count and age, depending on the vegetarian status. The general population (here abbreviated as omnivores (OMN)) is shown in red, whereas semi-vegetarians (SVG) are shown in blue and lacto-ovo-vegetarians (LOV) are shown in green. In panel a, we adjusted for age, sex and ethnicity. In addition to these 3 variables, panels b – h included additional adjustments. In panel i, we adjusted for all aforementioned covariates in a single model. None of these models revealed significant between group contrasts or differences from the grand mean in the vegetarian subgroups. While white blood cell counts tended to be lower in lacto-ovo-vegetarians, the CIs where quite large – probably owing to the small sample size of this subpopulation. When adjusting for body mass index (see panels c and i), no differences were seen in predicted means. Neither the contrasts from the reference level (non-vegetarians) nor from the grand mean were significant in any model.

In a similar style, Supplementary Fig. 11 displays plots of marginal predicted values (adjusted means) for the platelet count. Supplementary Figs. 12 and 13 show plots of marginal predicted values (adjusted means) for the lymphocyte and neutrophil count, respectively. Neutrophils showed a similar pattern as white blood cells and tended to be lower in lacto-ovo-vegetarians. Again, the CIs were quite large (likely owing to the small sample size of this subpopulation) and the between group differences were not significant.

**Fig. 2:**
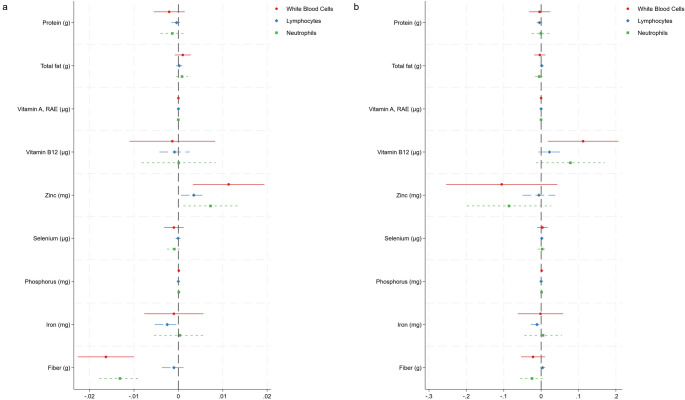
Coefficient plot—plotting regression coefficients from a multivariable regression model to predict white blood cell counts, lymphocyte counts and neutrophil counts. The entire sample is depicted on the left (panel **a**), whereas the vegetarian subsample is shown on the right (panel **b**, *n* = 208). In the entire sample, we found significant beta-coefficients for dietary fiber and zinc after adjustment for age, sex, race/ethnicity and smoking (coefficients not shown in this graphic). In the vegetarian subsample, we found a significant beta-coefficient for vitamin B12

All dietary variables of interest were then again added in a new set of multivariable linear regression models to predict white blood cell counts (and, in 3 separate models, platelet, lymphocyte and neutrophil counts) in the entire sample and in the self-identified vegetarian sample. Using Jann’s “coefplot”, we plotted the regression coefficients in Fig. 2 (for the white blood cell, lymphocyte and neutrophil count) and Supplementary Fig. 14 (platelet count). In the entire sample, each additional gram of dietary fiber was associated with a significant decrease in the white blood cell count by almost 0.02*1000 cells/µl and in the neutrophil count by 0.01*1000 cells/µl (panel a). Significant positive beta coefficients were also found for zinc. In the vegetarian subsample, each additional µg of dietary vitamin B12 was associated with an increase in white blood cells in vegetarians by 0.11*1000 cells/µl (panel b). A similar trend for platelets and fiber was found in the entire sample and the vegetarian subsample (Supplementary Fig. 14).

## Discussion

The present study investigated whole blood count data in US vegetarians in the NHANES (2007–2010). While crude and adjusted white blood cell counts were lower in lacto-ovo-vegetarians, they did not differ significantly from omnivores. Significant between group differences were found for the red blood cell count, the hematocrit and for hemoglobin levels. From a clinical point of view, differences were marginal except for hemoglobin levels. Several associations with nutrient intake data were found, including a significant correlation of the white blood cell counts with the BMI and selenium intakes in lacto-ovo-vegetarians (*r* = 0.27 and *r* = 0.27, respectively). In the entire sample, each additional gram of dietary fiber was associated with a decrease in the white blood cell count by almost 0.02*1000 cells/µl and in the neutrophil count by approximately 0.01*1000 cells/µl.

Our findings do not align with previous studies in other plant-based populations and thus warrant a thorough discussion [3, 5]. Studies by Haddad et al. and Tong et al. reported significantly lower white blood cell counts in American and British vegans/vegetarians in comparison to omnivores [3, 5]. We also observed lower white blood cell counts in lacto-ovo-vegetarians, however, the between group difference was much smaller than expected and the obtained estimates were not significant. A reservation must be made, that the herein examined unweighted lacto-ovo-vegetarian sample was rather small (*n* = 98) and the corresponding CIs were thus quite large.

Haddad et al. reported a white blood cell difference of approximately 1*10^9^/L between vegans (4.96 ± 0.91) and non-vegans (5.83 ± 1.51) [3]. Mean white blood cell counts in vegetarians in our sample were substantially higher (6.97*1000/µl) but quantitatively comparable with the data reported by Tong et al. based on British vegetarians [5]. One crucial factor that could explain our non-significant results is the average BMI of the examined participants. The mean BMI in vegetarians in this sample was 25.80 kg/m^2^, whereas Haddad et al. examined young, lean and healthy university students with a mean BMI of 20.5 kg/m^2^ [3]. A mean BMI of approximately 26 kg/m^2^ has been reported earlier for US vegetarians by Tonstad et al., and our findings were thus considered plausible and realistic [39]. Of note, correlations between the white blood cell count and the BMI were found in this and many other studies [40, 41]. Obesity and the subsequent overexpression of adipokines is known to promote chronic systemic inflammation and tissue damage [42, 43], and while vegetarians had a significantly lower BMI compared to their omnivorous counterparts in our study, they were still in the overweight range (25.00–29.99 kg/m^2^). According to the investigations by Tonstad et al., only one plant-based diet group was characterized by a normal body weight in the US: the vegans [39]. The pathological BMI in lacto-ovo-vegetarians in our sample may be *the* most important factor that could explain why our estimations for the white blood cell counts (and also the neutrophil counts) were not in line with other studies.

It is also interesting to note that selenium intake correlated significantly with white blood cell counts in lacto-ovo-vegetarians in our study (*r* = 0.27, *p* = 0.022). While the literature with regard to selenium intakes and white blood cell counts is inconsistent, there is some evidence suggesting that increasing selenium intake may increase white blood cell counts and activate human leukocytes [44–46]. Selenium intake and serum levels are usually lower in vegetarians and vegans when compared to omnivores [47, 48]. In our cohort, vegetarians had an average selenium intake of approximately 92 µg/d; a value which is untypically high for vegetarians in light of the reported intakes in other non-US-based vegetarian cohorts [48, 49]. Whether this could have contributed to the higher levels of white blood cells in vegetarians in this study remains subject to speculation.

Platelets also play an important role in immune-mediated inflammatory diseases [23, 50]. Platelets communicate with neutrophils, monocytes and various lymphocyte subsets, which allows them to actively modulate innate and adaptive immune responses [50]. Higher platelet levels and lower volumes in vegetarians have been reported previously in British white vegetarians and Chilean vegetarians [5, 51]. The herein presented results are largely in line with these findings, as reflected by the (non-significant) higher crude platelet counts in lacto-ovo-vegetarians. In BMI-adjusted models, marginal predicted platelet values were also found to be higher in lacto-ovo-vegetarians than in omnivores. Overall, higher platelet counts in vegetarians still appear surprising, since they might be an indicator of inflammation, whereas vegetarian diets might exert anti-inflammatory properties. To the best of our knowledge, the reasons for this phenomenon remain unknown.

Fiber intake could play an important role regarding platelet counts, given the observed significant inverse correlation with platelet counts in the lacto-ovo-vegetarian subgroup (*r* = − 0.23, *p* = 0.007). Notably, mean fiber intakes in vegetarians in this study were still below the nationally recommended fiber intakes (14 g/1000 kcal), which could have contributed to the aforementioned findings. The role of other macronutrients and micronutrients on platelet function has been discussed in detail elsewhere [52].

Lower cell counts in vegans and vegetarians are not univocally regarded as the result of the respective diets’ anti-inflammatory potential [5]. To the contrary, low white blood cell counts have been suggested as an indicator for an impaired immune system or bone marrow impairment [5], e.g., due to diet-induced nutritional deficiencies, including vitamin B12 or zinc [3, 9].

Vitamin B12 is a critical nutrient in all plant-based diets [53, 54], and the lower cell counts in vegetarians and vegans have been frequently attributed to a suboptimal or deficient B12 intake, which plays a crucial role in the maturation of red blood cells and the platelet life cycle [5, 55]. A suboptimal vitamin B12 status is frequently encountered in unsupplemented vegetarians and vegans [48], and should thus be considered in the context of hemogram alterations in these populations. Regrettably, serum vitamin B12 levels were not available for the 2007/2008 and 2009/2010 NHANES cycles [56], and we thus were only able to consider dietary intakes in our analyses. Vitamin B12 intake was significantly lower in vegetarians than in omnivores and, as expected, lowest in the lacto-ovo-vegetarian subpopulation (daily intake: 3.44 (µg/d)). While not correlated with white blood cell counts, a significant moderate positive correlation (*r* = 0.37, *p* = 0.030) between the vitamin B12 intake and the neutrophil count was found in semi-vegetarians. The lack of serum biomarkers, however, does not allow for any conclusive statements with regard to this aspect.

Finally, it is important to emphasize that dietary intakes in NHANES vegetarians were not optimal for many nutrients [32]. While their diet was found to be healthier than the diet of NHANES omnivores [18], the NHANES vegetarian dietary pattern was still characterized by suboptimal intakes of several nutrients, including potassium, fiber and others [57]. The comparison to other vegetarian and vegan cohorts with a more optimal nutrient intake profile thus remains inherently difficult. Our findings thus reiterate that not all plant-based diets are created equal, and that a meticulous differentiation is warranted up to the nutrient intake level [2].

Strengths and limitations of this analysis must be thoroughly considered. While NHANES vegetarians are suitable for the purpose of this analysis, the vegetarian subgroup sample size was small. CIs are thus expectedly large with survey data, making it very difficult to find significant between group differences and small effect sizes. Several proportions describing the sample had to be flagged as unreliable as per the recent NCHS guidelines [35]. Extrapolating findings to the general population might thus not be advisable in this context. Error-prone simulation studies would have been required to perform a power analysis with survey data and survey weights [58]. Considering this and the fact that the number of self-identified vegetarians with a full data set was limited, we refrained from that step. The cohort definition and construction by itself was a tradeoff between maintaining an adequate unweighted sample size and including as many variables as possible. One example is alcohol intake which was dichotomized for this analysis based on a single variable. Using a 3 category-based approach (“current vs former vs never drinker”) would have allowed for additional insights but would also have diminished the vegetarian subpopulation sample size by another 20%. Thus, we decided against this approach. Another example is the consideration of a second dietary recall, which would have added to the strength of our observations but was not done for sample size considerations.

While the accuracy of some estimates must be regarded with caution, the herein presented data allows for important insights into whole blood count data in US vegetarians. The higher age categories (70–79, 80 + years) contained a limited number of unweighted observations (*n* = 19 and *n* = 23, respectively), limiting the accuracy of our estimates. The lack of serum parameters for several variables of interest (e.g., vitamin B12) and the lack of other variables previously associated with lower cell counts in vegans (e.g., branched-chain amino acid intakes and serum levels [4]) may be considered as additional limitations. The dataset stems from the years 2007–2010, and may not reflect changes in vegetarian diet patterns over the last years. Then again, this analysis has several strengths worth mentioning, including the underlying NHANES dataset, the rigorous analytical approach based on the most recent recommendations by Heeringa et al. [29], the consideration of numerous potential confounders (e.g., data on recent infections) and the differentiation between two different plant-based patterns (lacto-ovo-vegetarian vs semi-vegetarian).

Despite potential limitations, the present study suggests that a lacto-ovo-vegetarian diet as found in NHANES vegetarians was not associated with significant hemogram alterations and may not confer the same benefits (e.g., lower white blood cell and platelet counts) as previously reported with vegan diets [3–5]. Additional research in larger datasets and well-controlled clinical intervention studies is warranted to deepen the current understanding of hemogram and hematological alterations in distinctive plant-based dietary patterns. As for the examined NHANES data, machine learning methods could provide an additional avenue for future research.

## Conclusions

Previously described hemogram alterations in vegetarians in other cohorts could only partially be replicated in NHANES vegetarians. While the results were directionally consistent with previous studies, between group differences in white blood cell and neutrophil counts were not statistically significant when comparing NHANES vegetarians to the general population. Vegetarians were characterized by lower red blood cell counts and hemoglobin levels, however, differences in the former were marginal from a clinical point of view. Statistical and design-specific aspects as well the elevated BMI in the vegetarian subpopulation could serve as potential explanations.

## Supplementary Information

Below is the link to the electronic supplementary material.

Supplementary Material 1

## Data Availability

Data is publicly available online (https://wwwn.cdc.gov/nchs/nhanes/Defaµlt.aspx). The datasets used and analyzed during the current study are available from the corresponding author on reasonable request.

## Abbreviations

CI: Confidence Interval
LOV: Lacto-ovo-vegetarian
NCHS: National Center for Health Statistics
NHANES: National Health and Nutrition Examination Survey
SVG: Semi-vegetarian
OMN: Omnivores
UK: United Kingdom
US: United States
VCS: Volume, conductivity, scatter
