# Supplementary material for: Whole blood count data and hematological parameters in US vegetarians: a cross-sectional study
Source: Eur J Nutr. 2025 Sep 18;64(6):280. doi: 10.1007/s00394-025-03778-8 (PMC12446107; doi:10.1007/s00394-025-03778-8)
Supplement: Supplementary file 1 — Supplementary Material 1 [file 394_2025_3778_MOESM1_ESM.docx]

# Supplementary Figures

## Supplementary Figure 1

Supplementary Figure 1 title: Participant inclusion flowchart


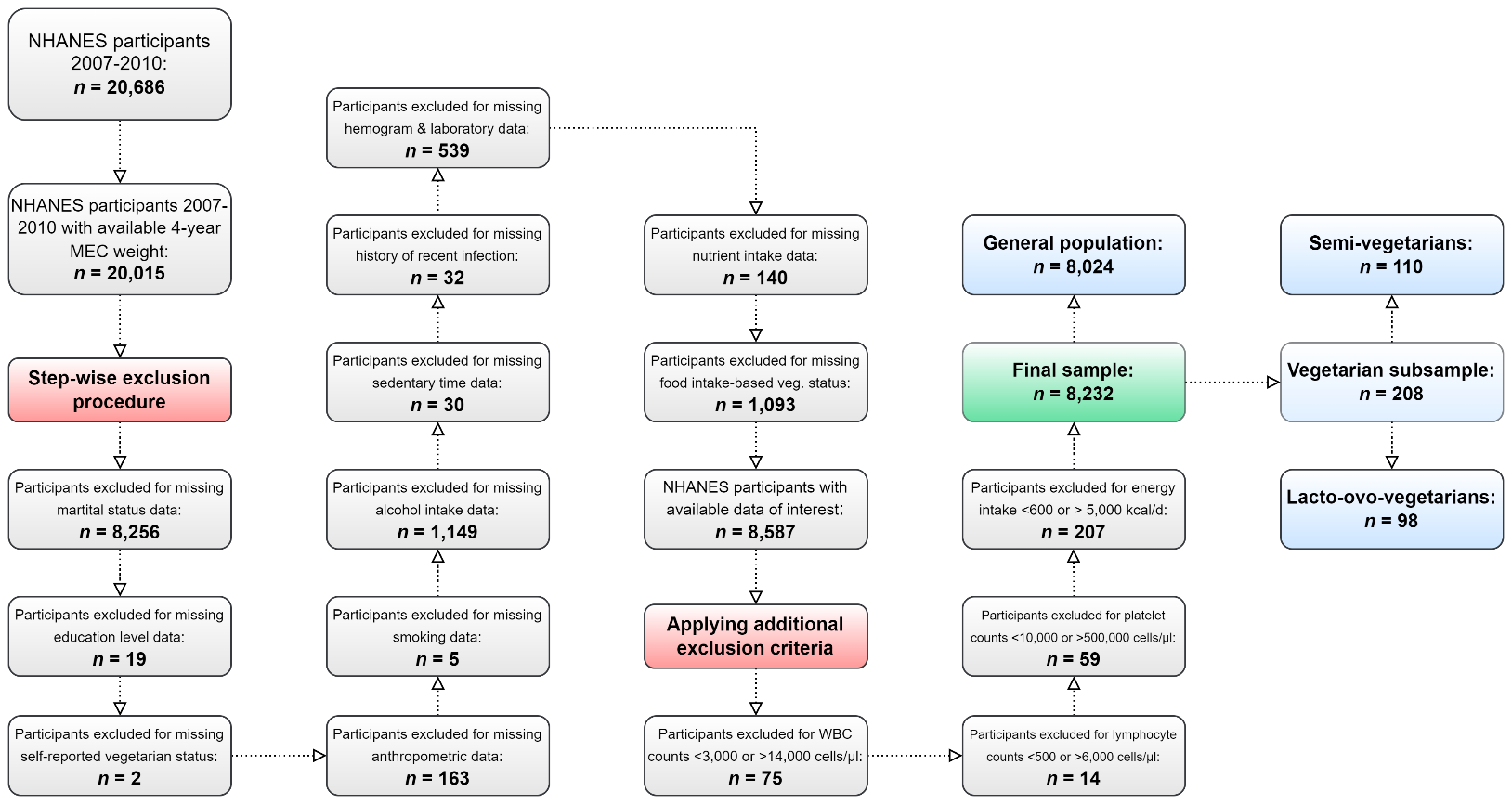


Supplementary Figure 1 legend: Supplementary Figure 1 depicts a participant inclusion flowchart with a step-by-step exclusion process. The total number of unweighted observations in this analysis was *n* = 8,232. The sample included *n* = 208 vegetarians, thereof *n* = 98 lacto-ovo-vegetarians and *n* = 110 semi-vegetarians.

## Supplementary Figure 2

Supplementary Figure 2 title: Histograms showing the distribution of the while blood cell count, lymphocyte count, neutrophil count and platelet count in the analyzed sample.


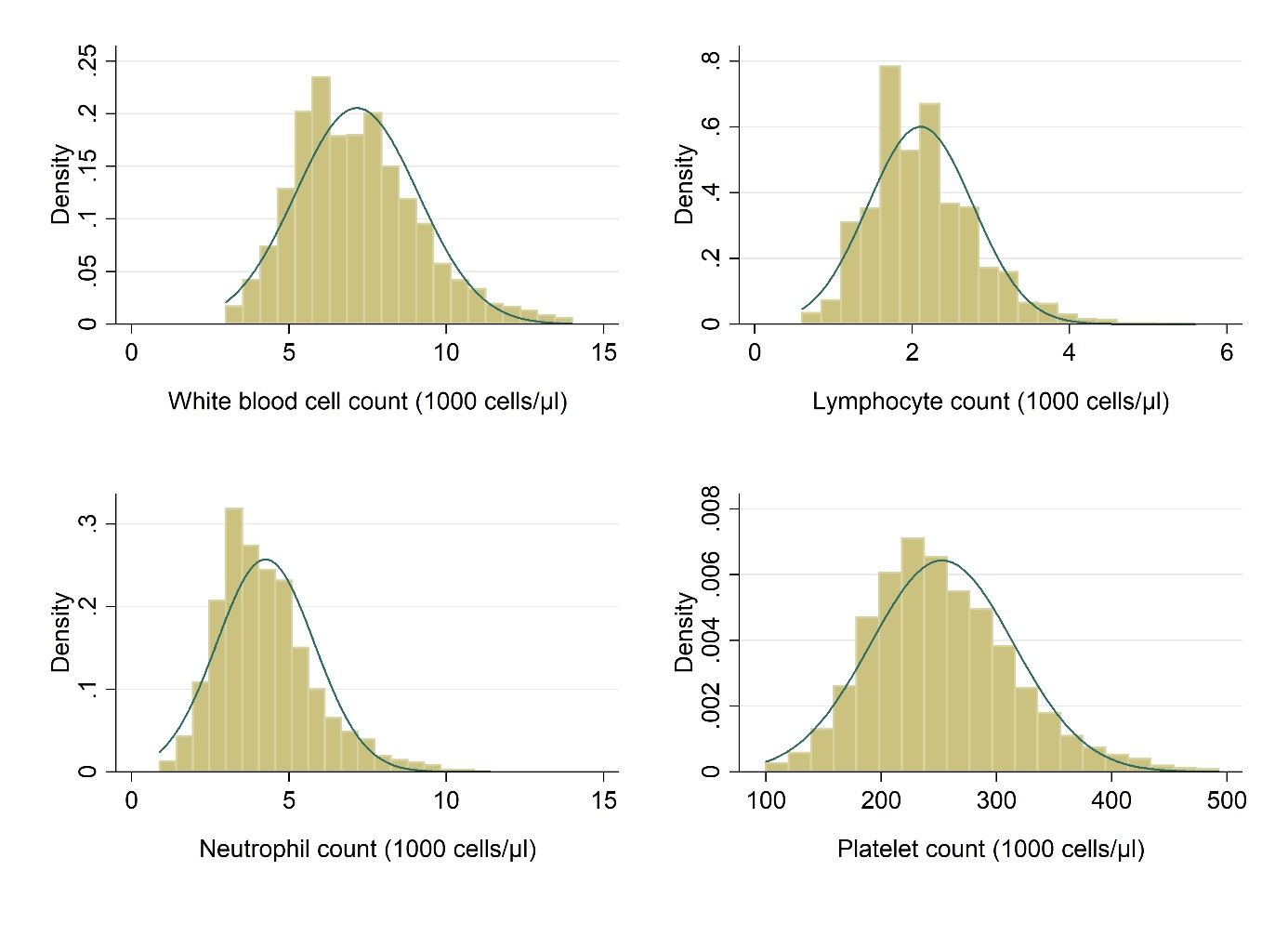


Supplementary Figure 2 legend: To create the histograms, we used the integer part of the sampling weight by creating a frequency weight from the sampling weight.

## Supplementary Figure 3

Supplementary Figure 3 title: Scatterplots of the white blood cell count (in 1000 cells/μl) and various other anthropometric (panel a: body mass index) and dietary variables (panels b-i).


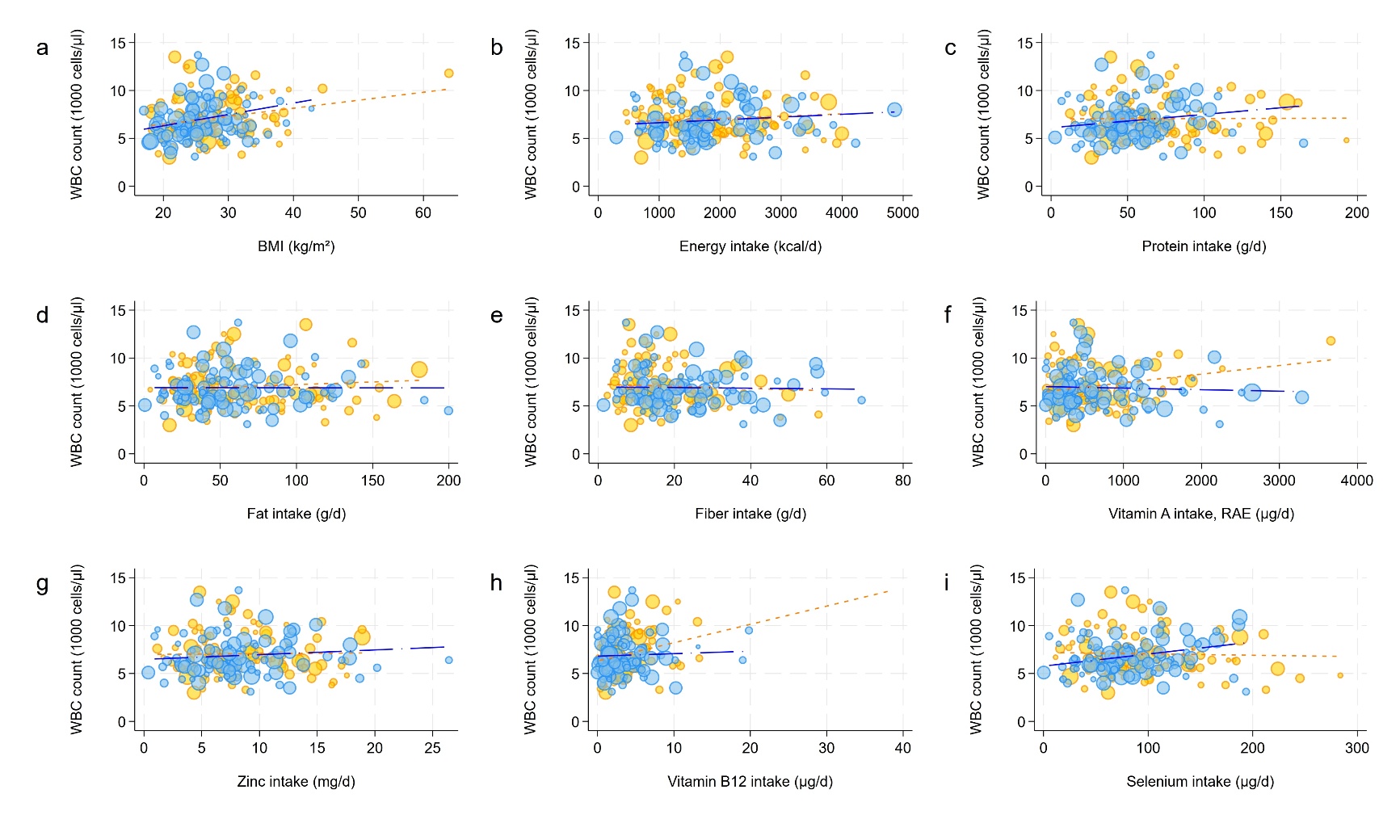


Supplementary Figure 3 legend: Weak but significant positive correlations were found for the body mass index (in kg/m²) and the white blood cell count (in 1000 cells/μl) in lacto-ovo-vegetarians (*r* = 0.27, *p* = 0.022) as well as for dietary selenium intake (in μg/d) and the white blood cell count (*r* = 0.27, *p* = 0.045). The scatterplots only depict the vegetarian subpopulation, whereby lacto-ovo-vegetarians are shown in blue (*n* = 98) and semi-vegetarians are shown in orange (*n* = 110).

## Supplementary Figure 4

Supplementary Figure 4 title: Scatterplots of the platelet count (in 1000 cells/μl) and various other anthropometric (panel a: body mass index) and dietary variables (panels b-i).


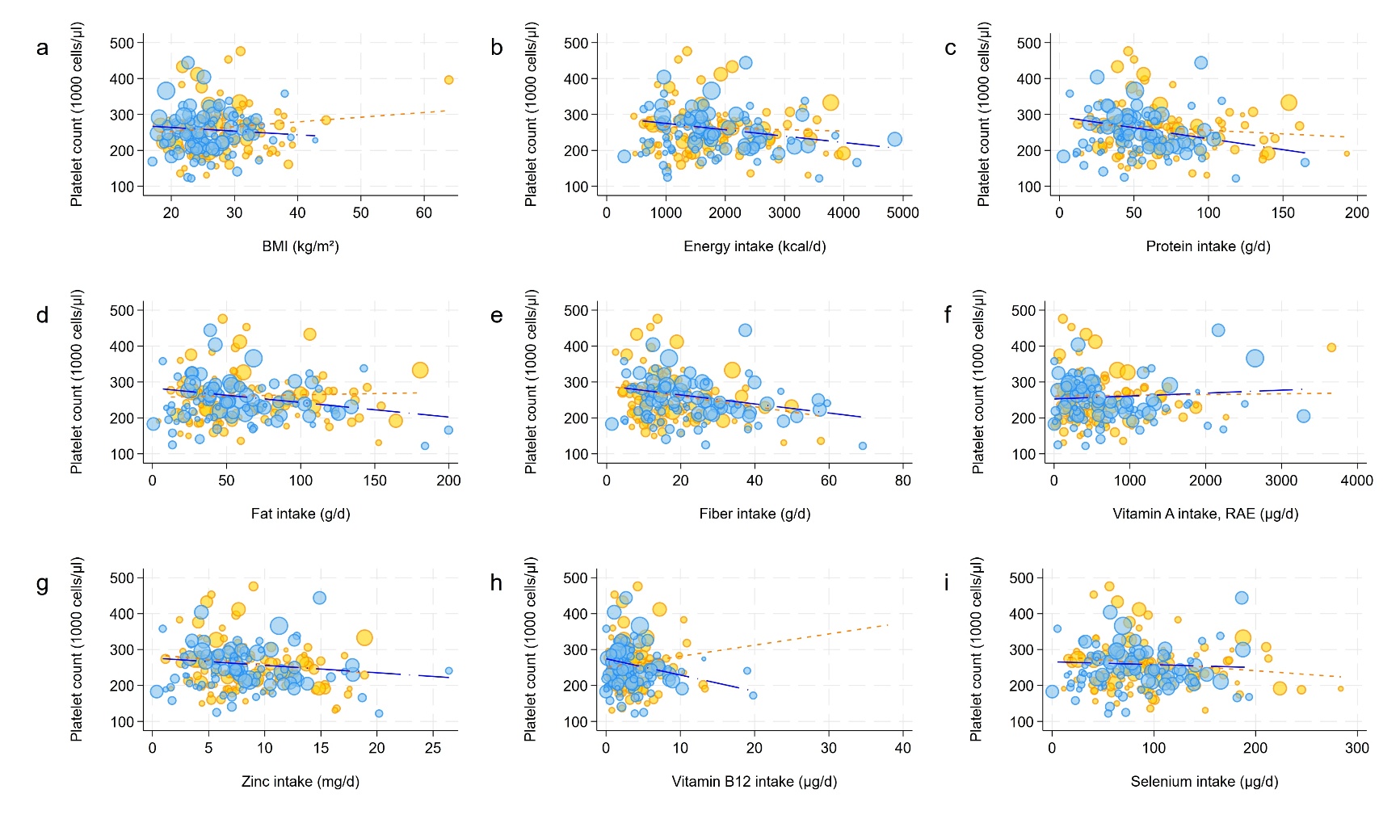


Supplementary Figure 4 legend: Weak but significant inverse correlations were found for the energy intake (in kcal/d) and the platelet count (in 1000 cells/μl) in lacto-ovo-vegetarians (*r* = -0.20, *p* = 0.001) as well as the fiber intake (in g/d) and the platelet count (in 1000 cells/μl) (*r* = -0.23, *p* = 0.007). Additionally, a weak significant inverse correlation was found for the protein intake and the vitamin B12 intake (in μg/d) and the platelet count (in 1000 cells/μl) in lacto-ovo-vegetarians (*r* = -0.20, *p* = 0.016 and *r* = -0.19, *p* = 0.038, respectively). In semi-vegetarians, a comparable inverse association between fiber intake and the platelet count was found (*r* = -0.23, *p* = 0.023). The scatterplots only depict the vegetarian subpopulation, whereby lacto-ovo-vegetarians are shown in blue (*n* = 98) and semi-vegetarians are shown in orange (*n* = 110).

## Supplementary Figure 5

Supplementary Figure 5 title: Scatterplots of the lymphocyte count (in 1000 cells/μl) and various other anthropometric (panel a: body mass index) and dietary variables (panels b-i).


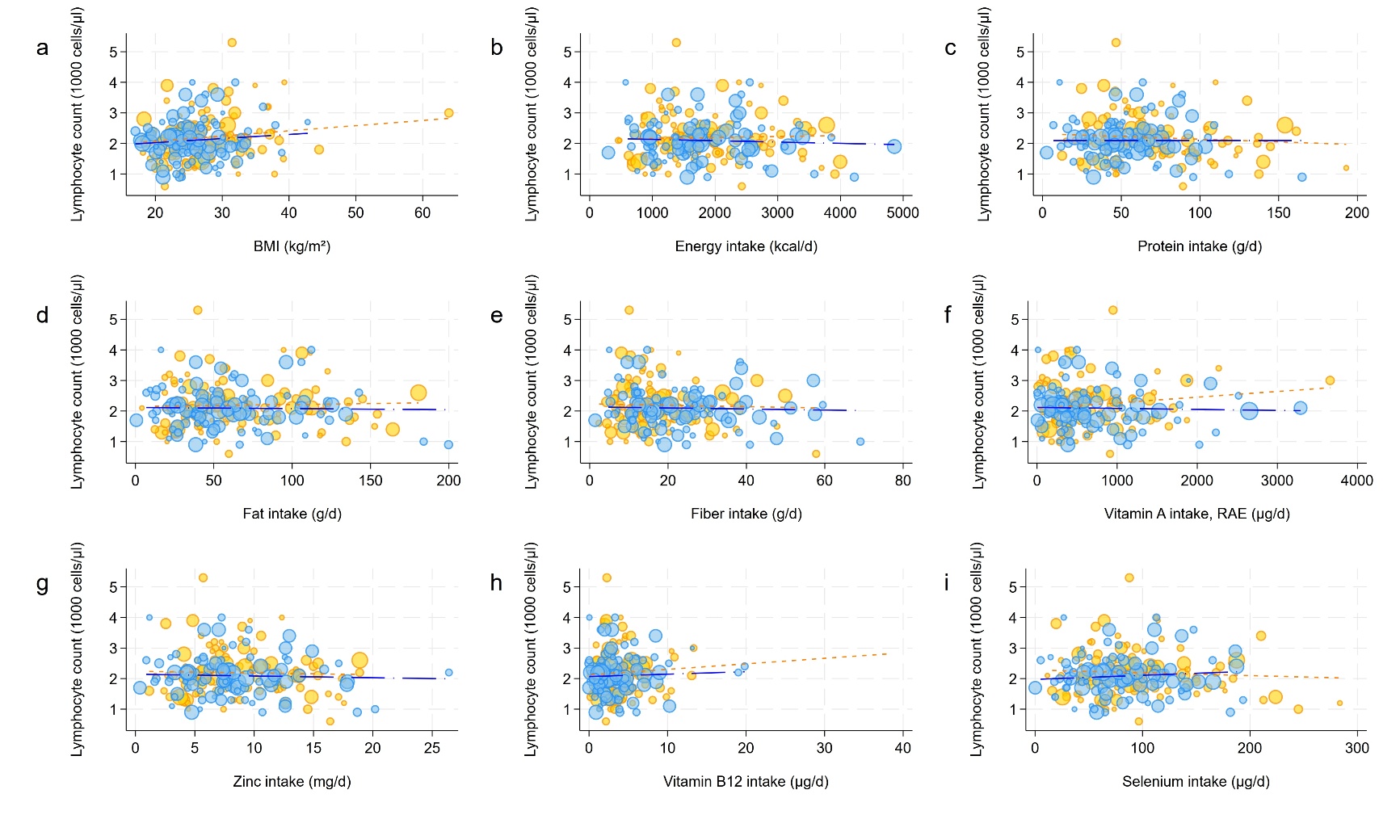


Supplementary Figure 5 legend: No significant associations were found. The scatterplots only depict the vegetarian subpopulation, whereby lacto-ovo-vegetarians are shown in blue (*n* = 98) and semi-vegetarians are shown in orange (*n* = 110).

## Supplementary Figure 6

Supplementary Figure 6 title: Scatterplots of the segmented neutrophil count (in 1000 cells/μl) and various other anthropometric (panel a: body mass index) and dietary variables (panels b-i).


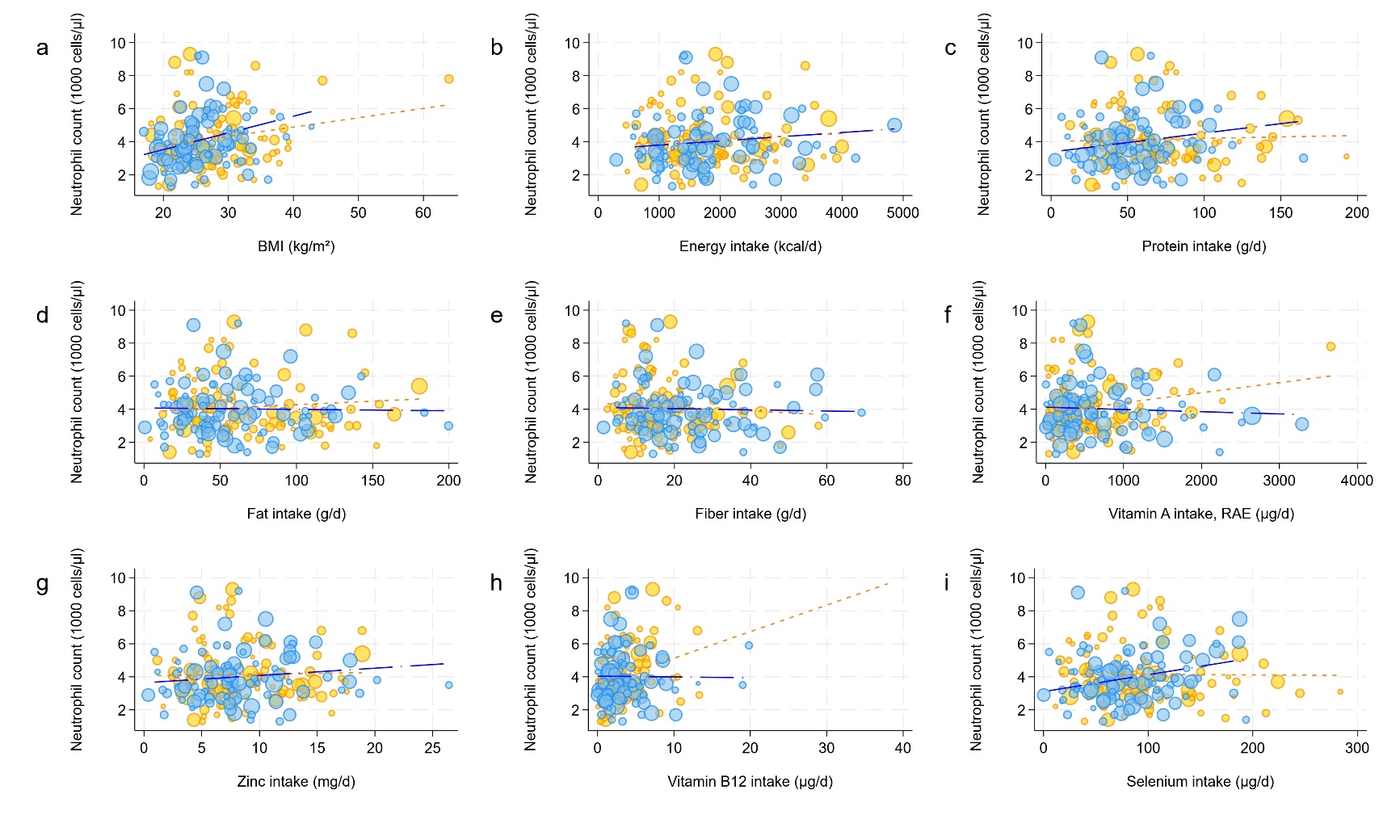


Supplementary Figure 6 legend: A weak but significant positive correlation was found for the body mass index (in kg/m²) and the neutrophil count (in 1000 cells/μl) in lacto-ovo-vegetarians (*r* = 0.29, *p* = 0.012). In semi-vegetarians, we found a moderate positive correlation between the vitamin B12 (in μg/d) intake and the neutrophil count (*r* = 0.37, *p* = 0.03). The scatterplots only depict the vegetarian subpopulation, whereby lacto-ovo-vegetarians are shown in blue (*n* = 98) and semi-vegetarians are shown in orange (*n* = 110).

## Supplementary Figure 7

Supplementary Figure 7 title: Scatterplots of the white blood cell count (in 1000 cells/μl) and intakes of various dietary fats


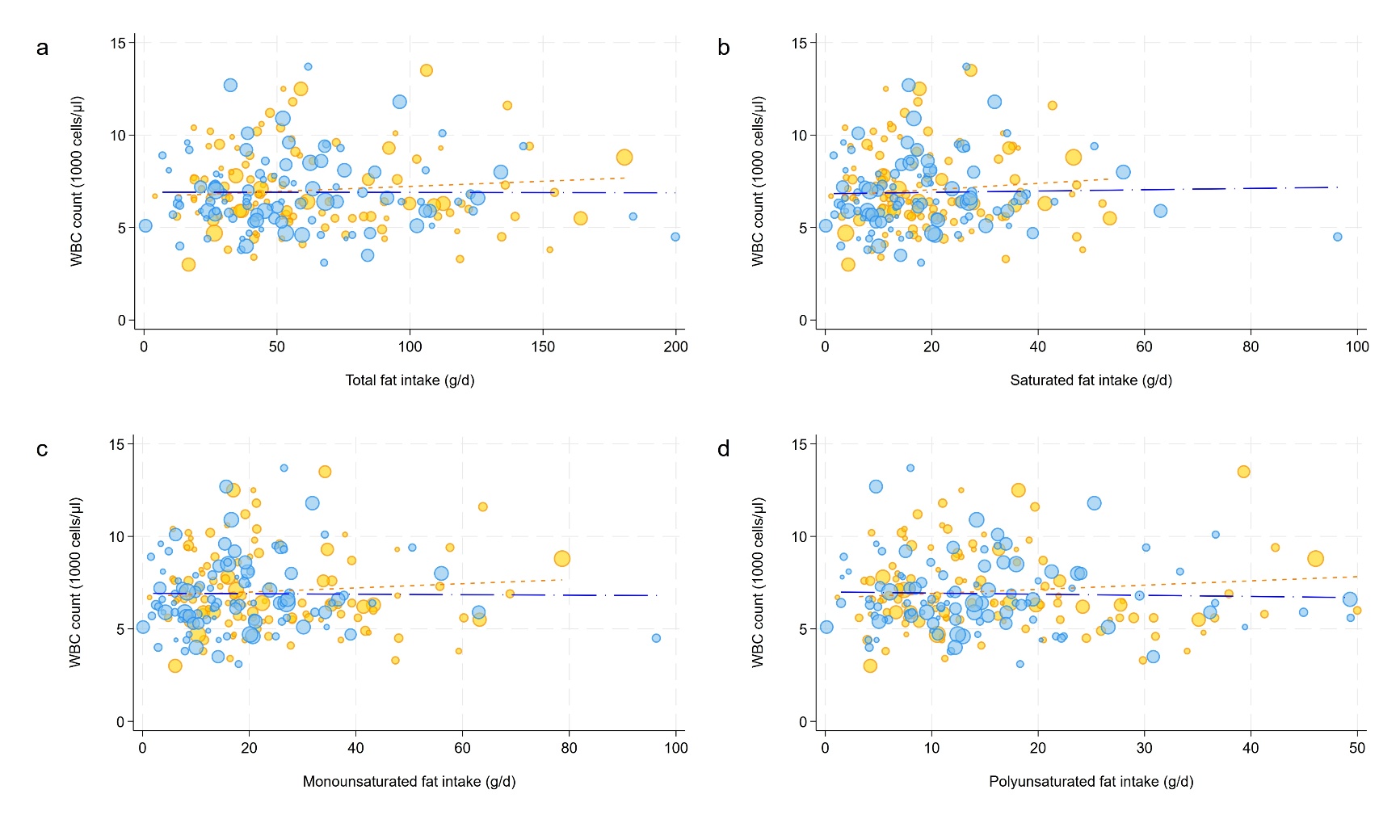


Supplementary Figure 7 legend: No significant associations were found between the white blood cell count and total fat intake (a), saturated fat intake (b), monounsaturated fat intake (c) and polyunsaturated fat intake (d). The scatterplots only depict the vegetarian subpopulation, whereby lacto-ovo-vegetarians are shown in blue (*n* = 98) and semi-vegetarians are shown in orange (*n* = 110).

## Supplementary Figure 8

Supplementary Figure 8 title: Scatterplots of the platelet count (in 1000 cells/μl) and intakes of various dietary fats


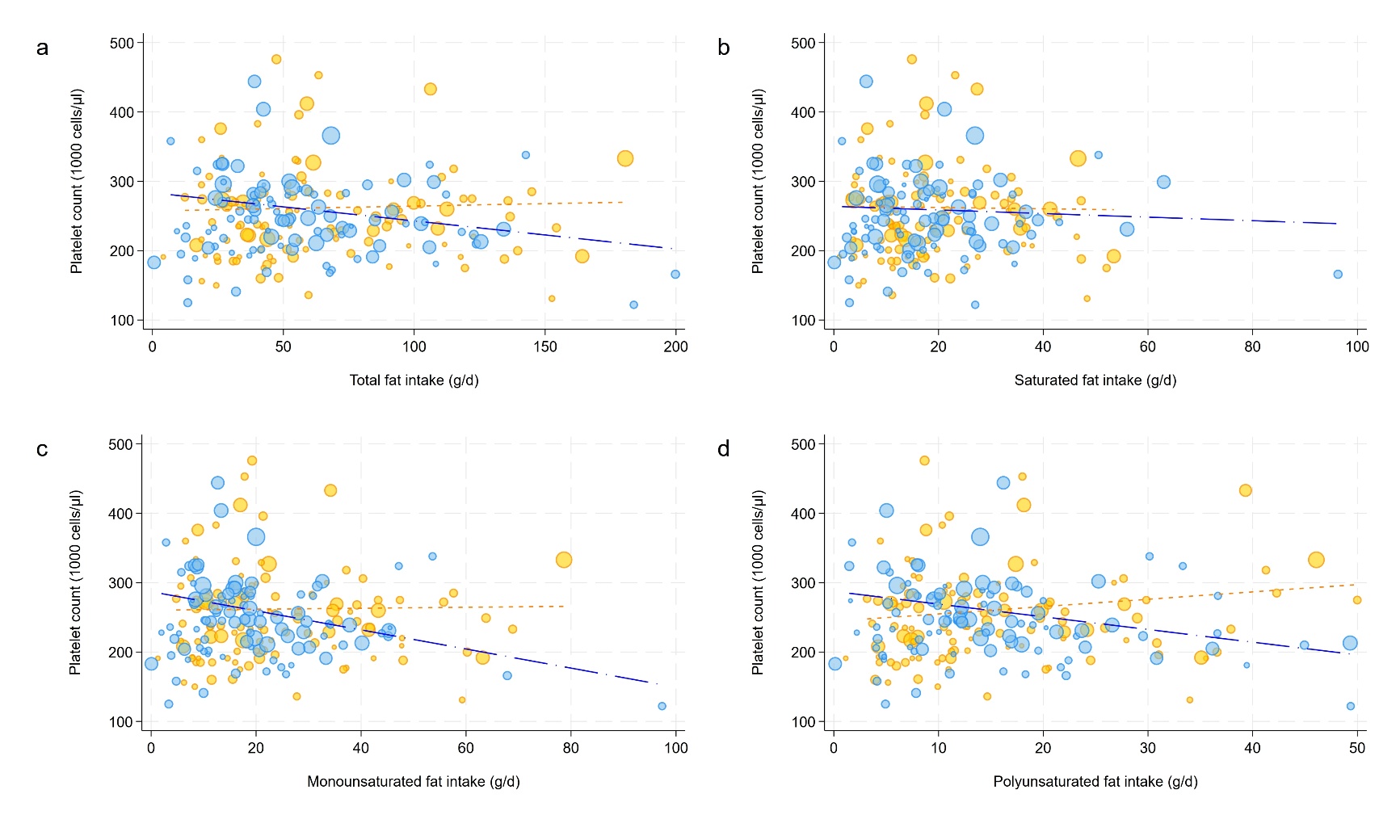


Supplementary Figure 8 legend: No significant associations were found between the saturated fat intake and the platelet count (b). Significant but weak inverse correlations were found for total fat intake in lacto-ovo-vegetarians (*r* = -0.19, *p* = 0.038), monounsaturated fat intake (*r* = -0.25, *p* = 0.018), and polyunsaturated fat intake (*r* = -0.26, *p* = 0.004). The scatterplots only depict the vegetarian subpopulation, whereby lacto-ovo-vegetarians are shown in blue (*n* = 98) and semi-vegetarians are shown in orange (*n* = 110).

## Supplementary Figure 9

Supplementary Figure 9 title: Scatterplots of the lymphocyte count (in 1000 cells/μl) and intakes of various dietary fats


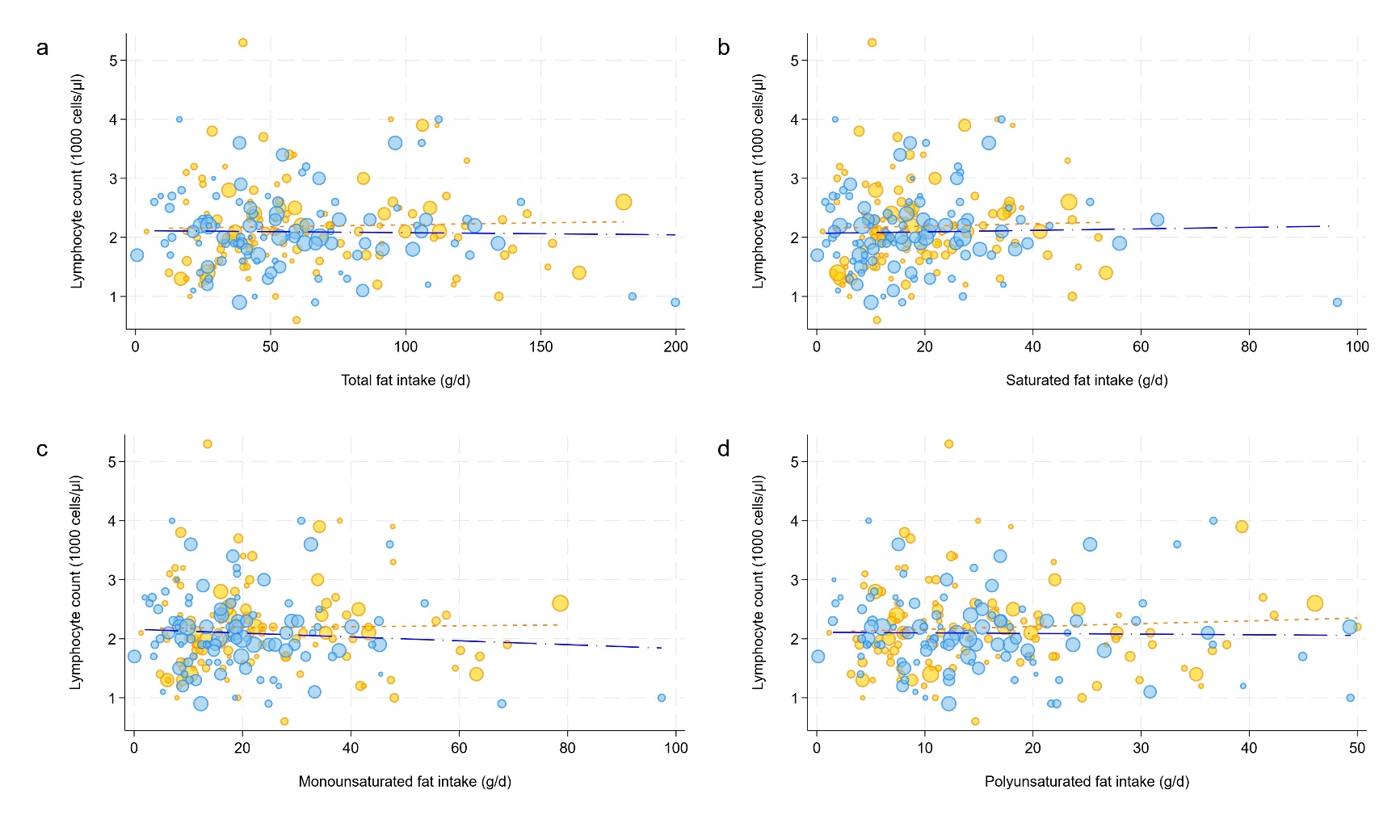


Supplementary Figure 9 legend: No significant associations were found between the lymphocyte count and total fat intake (a), saturated fat intake (b), monounsaturated fat intake (c) and polyunsaturated fat intake (d). The scatterplots only depict the vegetarian subpopulation, whereby lacto-ovo-vegetarians are shown in blue (*n* = 98) and semi-vegetarians are shown in orange (*n* = 110).

## Supplementary Figure 10

Supplementary Figure 10 title: Scatterplots of the neutrophil count (in 1000 cells/μl) and intakes of various dietary fats


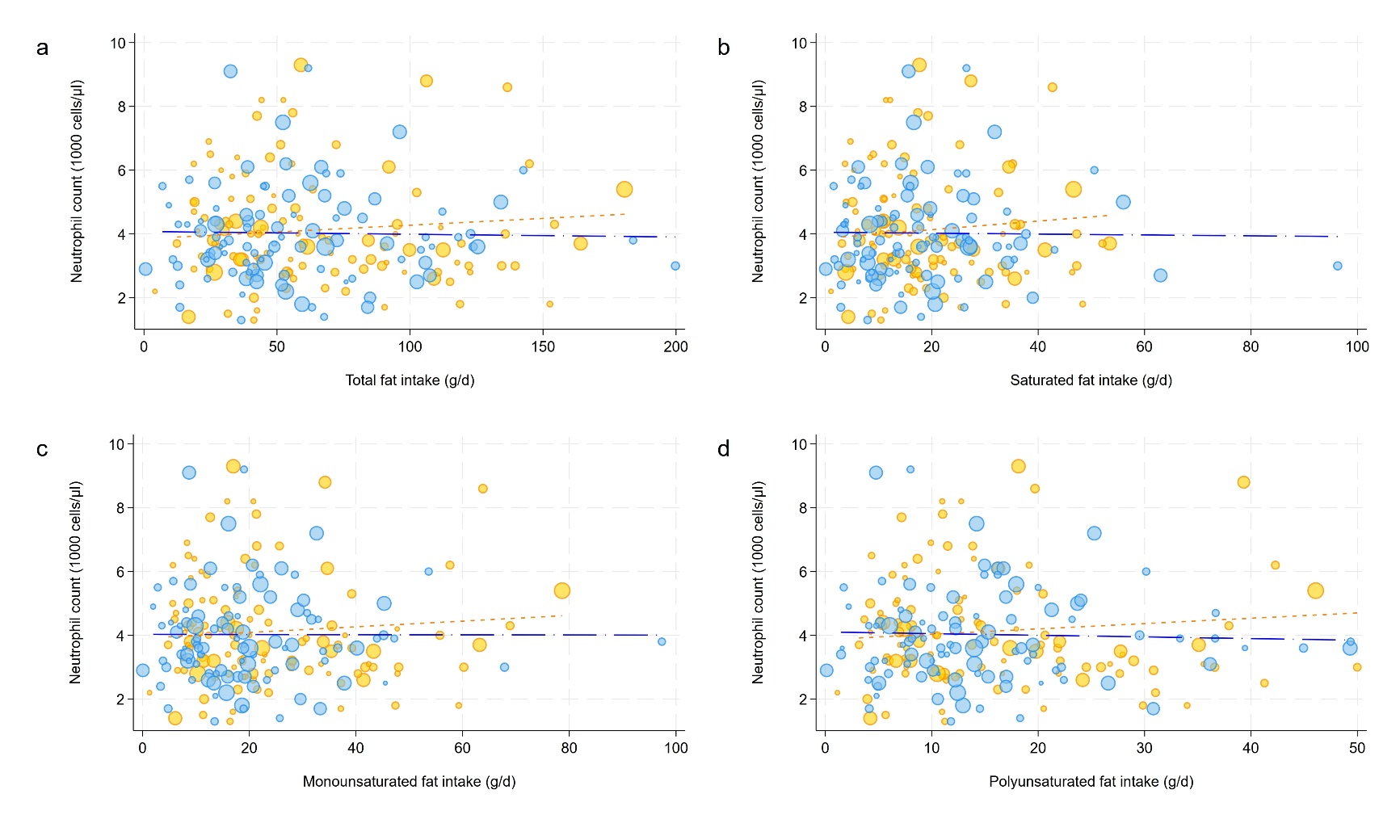


Supplementary Figure 10 legend: No significant associations were found between the neutrophil count and total fat intake (a), saturated fat intake (b), monounsaturated fat intake (c) and polyunsaturated fat intake (d). The scatterplots only depict the vegetarian subpopulation, whereby lacto-ovo-vegetarians are shown in blue (*n* = 98) and semi-vegetarians are shown in orange (*n* = 110).

## Supplementary Figure 11

Supplementary Figure 11 title: Margins plot – platelet count by vegetarian status


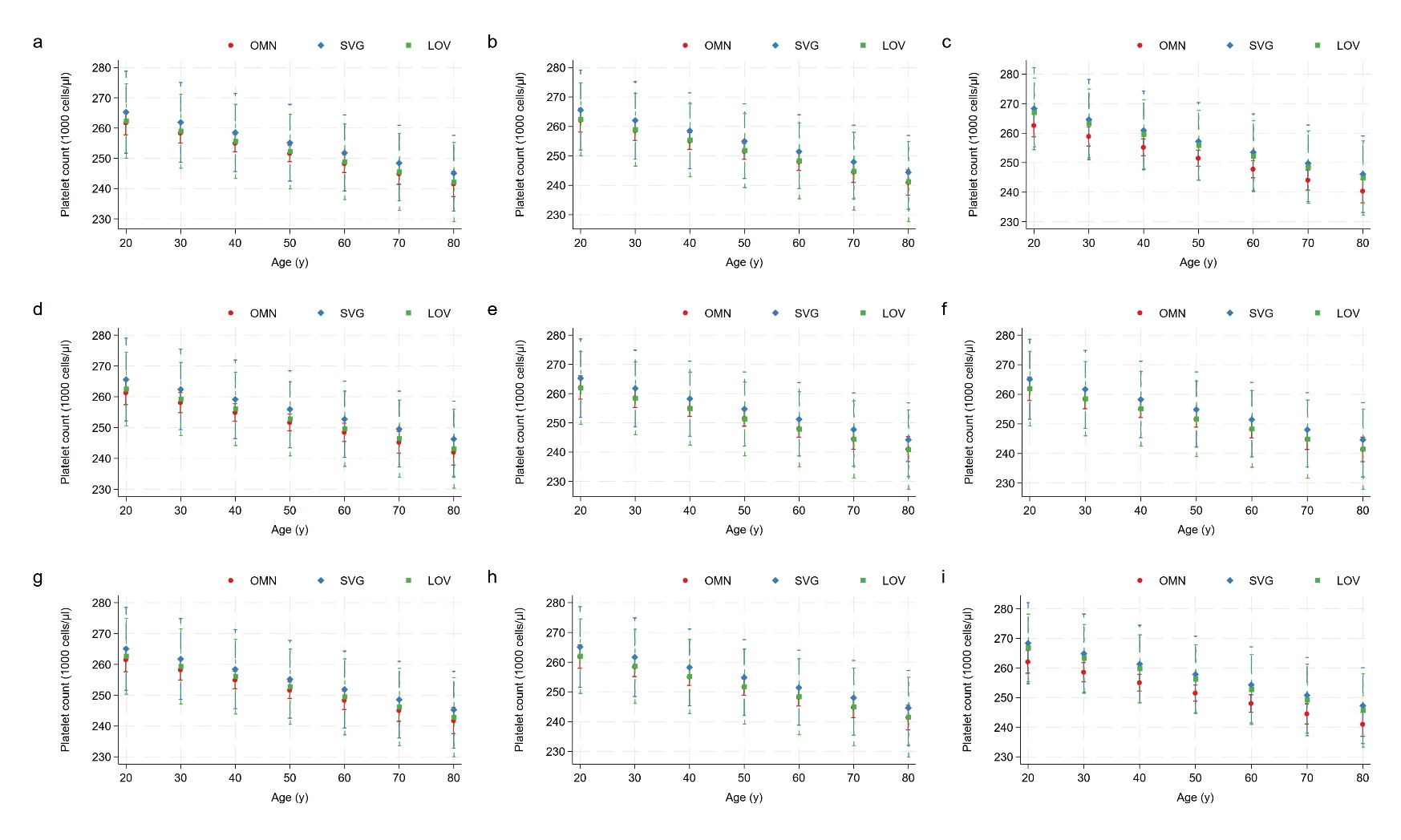


Supplementary Figure 11 legend: Plots of marginal predicted values (adjusted means) based on a series of regression models, illustrating differences in the relationship of the platelet count (in 1000 cells/μl) and age, depending on the vegetarian status. The general population (OMN – omnivores) is shown in red, whereas semi-vegetarians (SVG) are shown in blue and lacto-ovo-vegetarians (LOV) are shown in green. In panel a, we adjusted for age, sex and ethnicity. In addition to these 3 variables, panels b – h included the following adjustments: b: additional adjustment for energy intake (kcal/d); c: additional adjustment for the body mass index (kg/m²), d: additional adjustment for alcohol intake and smoking status; e: additional adjustment for selenium intake; f: additional adjustment for vitamin B12 intake; g: additional adjustment for vitamin A intake; additional adjustment for zinc intake. In panel i, we adjusted for all aforementioned covariates in a singular model.

## Supplementary Figure 12

Supplementary Figure 12 title: Margins plot – lymphocyte count by vegetarian status


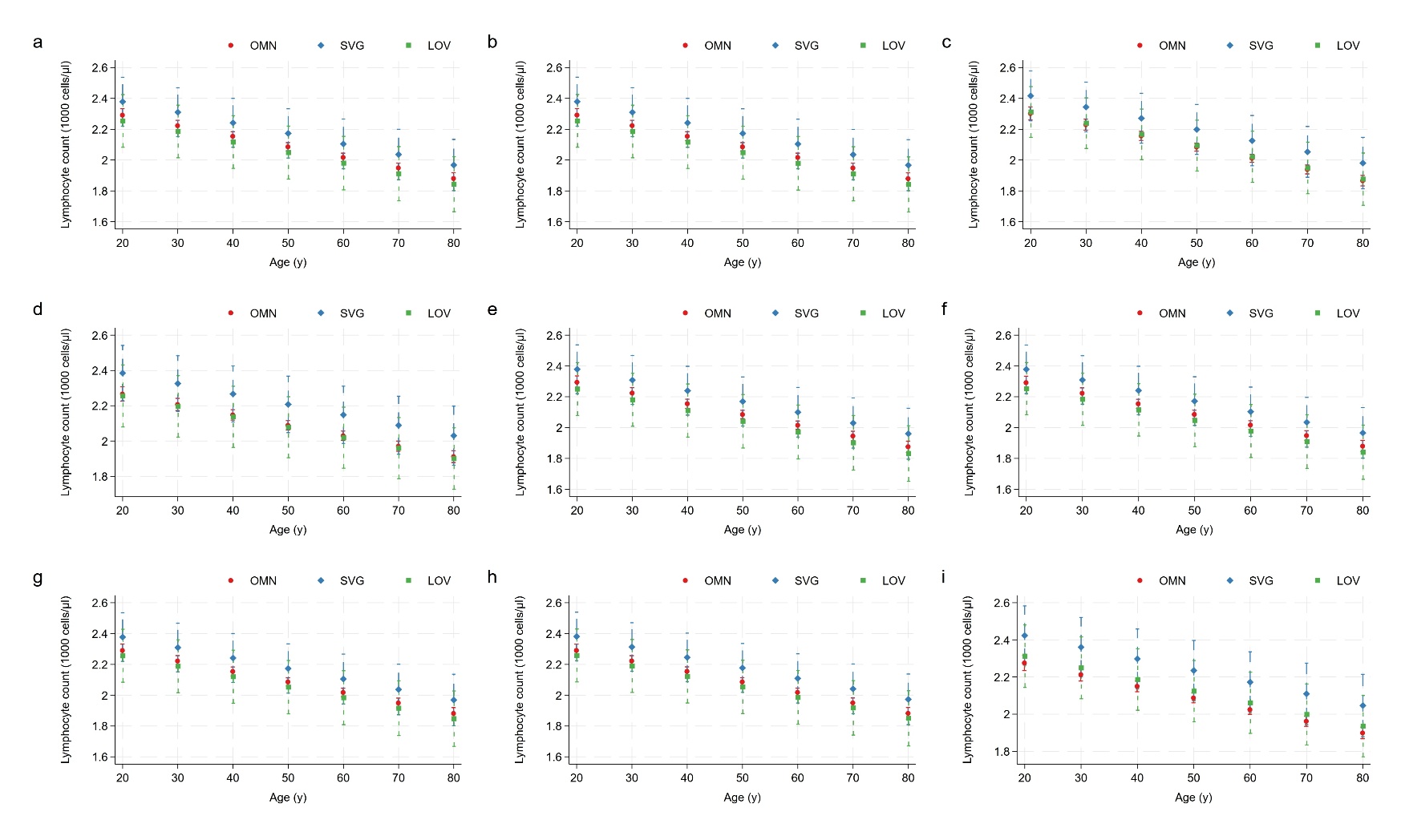


Supplementary Figure 12 legend: Plots of marginal predicted values (adjusted means) based on a series of regression models, illustrating differences in the relationship of the lymphocyte count (in 1000 cells/μl) and age, depending on the vegetarian status. The general population (OMN – omnivores) is shown in red, whereas semi-vegetarians (SVG) are shown in blue and lacto-ovo-vegetarians (LOV) are shown in green. In panel a, we adjusted for age, sex and ethnicity. In addition to these 3 variables, panels b – h included the following adjustments: b: additional adjustment for energy intake (kcal/d); c: additional adjustment for the body mass index (kg/m²), d: additional adjustment for alcohol intake and smoking status; e: additional adjustment for selenium intake; f: additional adjustment for vitamin B12 intake; g: additional adjustment for vitamin A intake; additional adjustment for zinc intake. In panel i, we adjusted for all aforementioned covariates in a singular model.

## Supplementary Figure 13

Supplementary Figure 13 title: Margins plot – neutrophil count by vegetarian status


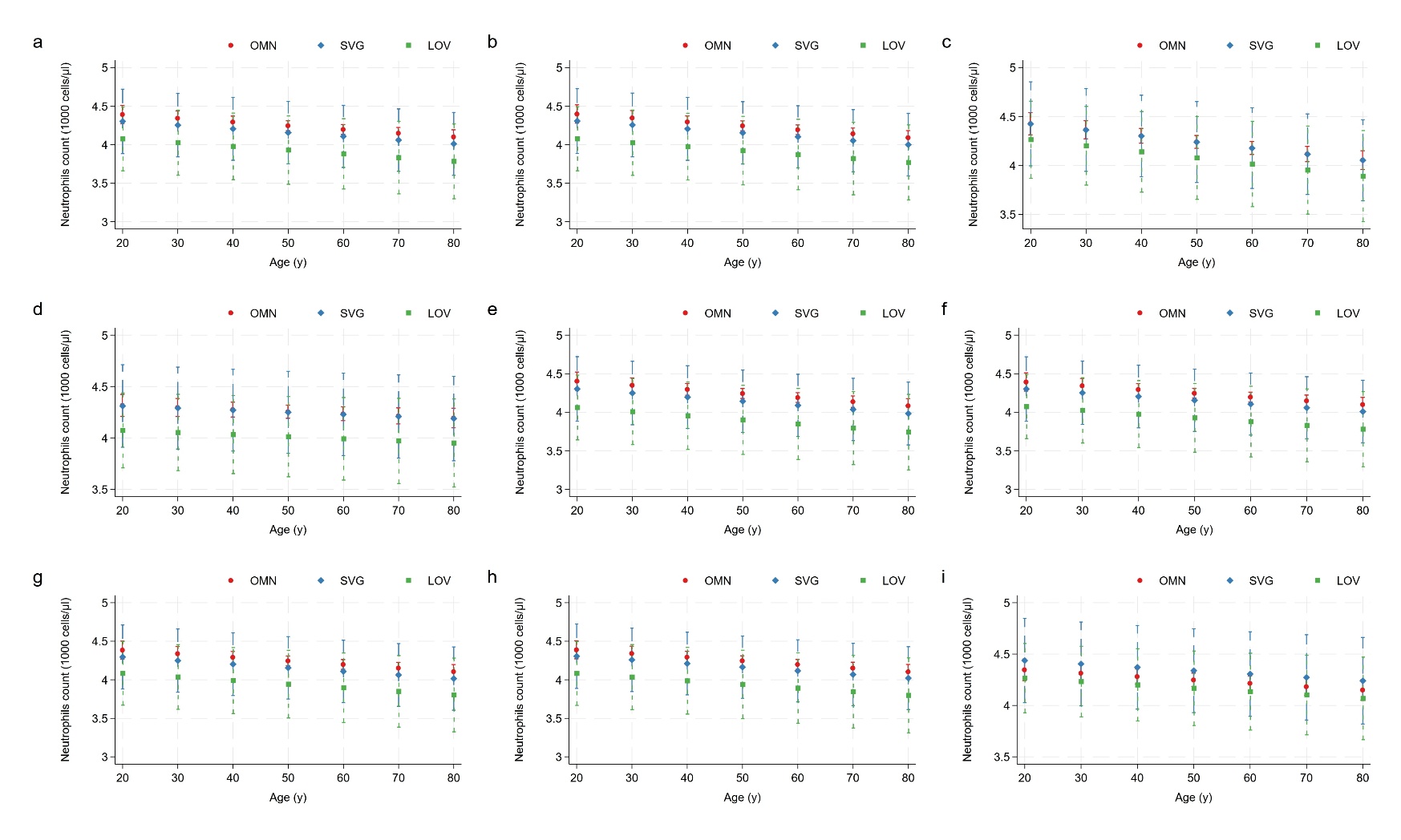


Supplementary Figure 13 legend: Plots of marginal predicted values (adjusted means) based on a series of regression models, illustrating differences in the relationship of the neutrophil count (in 1000 cells/μl) and age, depending on the vegetarian status. The general population (OMN – omnivores) is shown in red, whereas semi-vegetarians (SVG) are shown in blue and lacto-ovo-vegetarians (LOV) are shown in green. In panel a, we adjusted for age, sex and ethnicity. In addition to these 3 variables, panels b – h included the following adjustments: b: additional adjustment for energy intake (kcal/d); c: additional adjustment for the body mass index (kg/m²), d: additional adjustment for alcohol intake and smoking status; e: additional adjustment for selenium intake; f: additional adjustment for vitamin B12 intake; g: additional adjustment for vitamin A intake; additional adjustment for zinc intake. In panel i, we adjusted for all aforementioned covariates in a singular model.

## Supplementary Figure 14

Supplementary Figure 14 title: Coefficient plot - plotting regression coefficients from a multivariate regression model to predict platelet counts


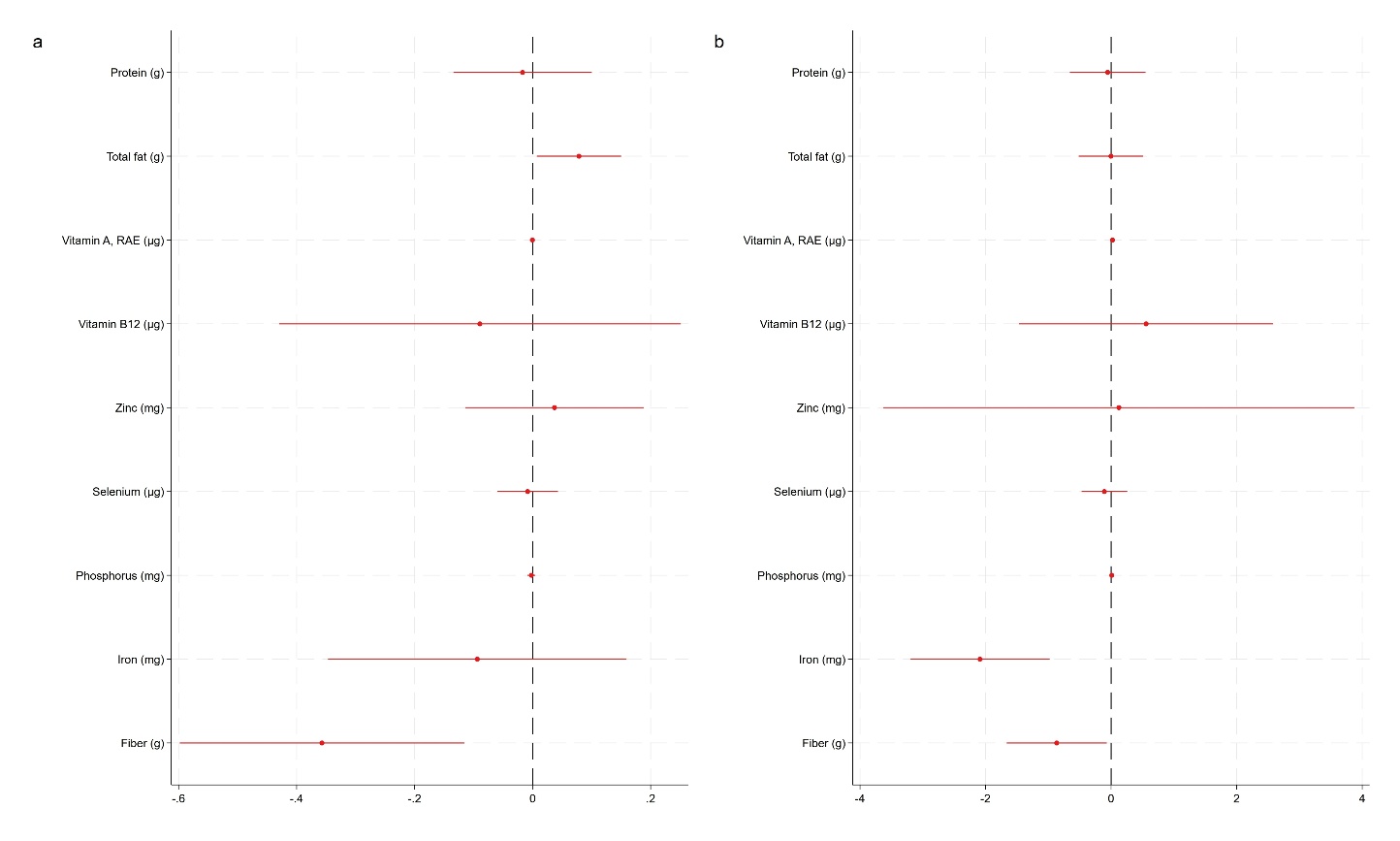


Supplementary Figure 14 legend: The entire sample is depicted on the left (panel a), whereas the vegetarian subsample is shown on the right (panel b, *n* = 208). In the entire sample, we found significant beta-coefficients for dietary fiber and fat after adjustment for age, sex, race/ethnicity and smoking (coefficients not shown in this graphic). In the vegetarian subsample, we found a significant beta-coefficient for fiber and iron.

END OF SUPPLEMENATARY DATA FILE
